# Quantification of folate metabolism using transient metabolic flux analysis

**DOI:** 10.1186/s40170-015-0132-6

**Published:** 2015-05-28

**Authors:** Philip M Tedeschi, Nadine Johnson-Farley, Hongxia Lin, Laura M Shelton, Takushi Ooga, Gillian Mackay, Niels Van Den Broek, Joseph R Bertino, Alexei Vazquez

**Affiliations:** Rutgers Cancer Institute of New Jersey, New Brunswick, NJ 08901 USA; Human Metabolome Technologies America, Boston, MA 02134 USA; Human Metabolome Technologies, Tsuruoka, Yamagata 997-0052 Japan; Cancer Research UK Beatson Institute, Garscube Estate, Switchback Road, Glasgow, G61 1BD UK

**Keywords:** Metabolic flux analysis, Folate metabolism, Methotrexate

## Abstract

**Background:**

Systematic quantitative methodologies are needed to understand the heterogeneity of cell metabolism across cell types in normal physiology, disease, and treatment. Metabolic flux analysis (MFA) can be used to infer steady state fluxes, but it does not apply for transient dynamics. Kinetic flux profiling (KFP) can be used in the context of transient dynamics, and it is the current gold standard. However, KFP requires measurements at several time points, limiting its use in high-throughput applications.

**Results:**

Here we propose transient MFA (tMFA) as a cost-effective methodology to quantify metabolic fluxes using metabolomics and isotope tracing. tMFA exploits the time scale separation between the dynamics of different metabolites to obtain mathematical equations relating metabolic fluxes to metabolite concentrations and isotope fractions. We show that the isotope fractions of serine and glycine are at steady state 8 h after addition of a tracer, while those of purines and glutathione are following a transient dynamics with an approximately constant turnover rate per unit of metabolite, supporting the application of tMFA to the analysis of folate metabolism. Using tMFA, we investigate the heterogeneity of folate metabolism and the response to the antifolate methotrexate in breast cancer cells. Our analysis indicates that methotrexate not only inhibits purine synthesis but also induces an increase in the AMP/ATP ratio, activation of AMP kinase (AMPK), and the inhibition of protein and glutathione synthesis. We also find that in some cancer cells, the generation of one-carbon units from serine exceeds the biosynthetic demand.

**Conclusions:**

This work validates tMFA as a cost-effective methodology to investigate cell metabolism. Using tMFA, we have shown that the effects of treatment with the antifolate methotrexate extend beyond inhibition of purine synthesis and propagate to other pathways in central metabolism.

**Electronic supplementary material:**

The online version of this article (doi:10.1186/s40170-015-0132-6) contains supplementary material, which is available to authorized users.

## Background

**Figure 1:**
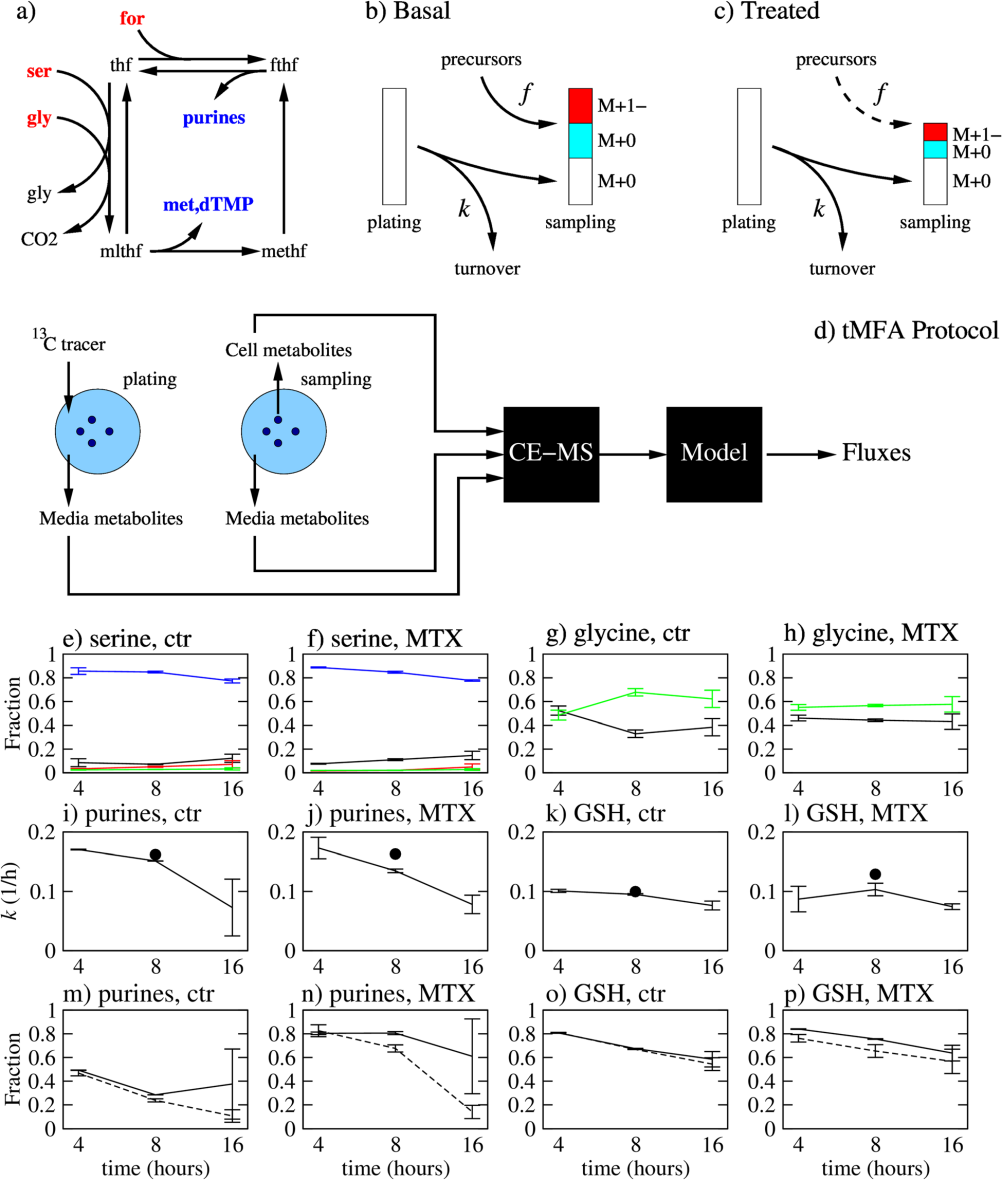
tMFA protocol and validation. **(a)** Simplified diagram of one-carbon metabolism. **(b)** Schematic representation of changes in isotope fractions after addition of a tracer in the culture media at plating. The white bars represent the unlabeled fraction from the intracellular metabolite present at plating, the cyan bar the unlabeled metabolite synthesized from unlabeled precursors and the red bar the labeled metabolite synthesized from labeled precursors. *f* denotes the rate of synthesis and *k* the rate of turnover per unit of metabolite. **(c)** Changes in the isotope pools due to inhibition of metabolite synthesis. **(d)** Sketch of the tMFA protocol. **(e**-**h)** Isotope fractions of serine and glycine in MCF7 cells, untreated or treated with MTX (black, M+0; red, M+1; green, M+2, and blue, M+3). **(i**-**l)** Estimated turnover rate of purines and glutathione (GSH) in MCF7 cells, untreated or treated with MTX (black lines, experiment 1, LC-MS quantification; circle, experiment 2, CE-MS quantification). The lines/points represent the average and the error bars the standard deviation over three replicates. **(m**-**p)** Comparison between the predicted isotope fractions based on turnover estimates for Exp 2 (dashed line) and the isotope fractions measured in Exp 1 (solid line), for both purines and glutathione in untreated and MTX-treated cells.

Folates are carriers of one-carbon units that are transferred between metabolites [1] (Figure 1a). Purines, thymidylate, and methionine are one-carbon acceptors required for cell proliferation. Serine, glycine, and formate are one-carbon donors, and their relevance in specific cellular systems is a current topic of intense research [2-5]. Serine can donate a one-carbon unit via the activity of serine hydroxymethyl transferase (SHMT), and glycine can donate one-carbon units via the glycine cleavage system (GCS). Yet, in some cancer cells, serine is the major one-carbon donor, they grow poorly in a serine-depleted medium, and this serine dependence cannot be rescued by supplementation of glycine in the culture medium [3].

Recent discoveries are pointing to new roles of folate metabolism beyond the balance of one-carbon units [4,6,7]. Theoretically, folate cycles can produce ATP via the activity of reverse formyl-tetrahydrofolate synthase (FTHFS). The ATP production is supported by flux balance modeling [6,7] and the induction of energy stress upon inhibition of folate metabolism [7]. The folate cycles can also produce NADPH via the activity of 5,10-methylene-tretrahydrofolate dehydrogenase (MTHFD) and/or 10-formyl-tetrahydrofolate dehydrogenase (FTHFD). The NADPH production is supported by flux balance modeling [4,6,7], and it has been experimentally demonstrated using ^3^H tracing [4]. When either reverse FTHFS or FTHFD is used to maintain the tetrahydrofolate (THF) balance, the one-carbon does not contribute to biosynthesis, but it is released as formate or CO_2_, respectively.

The heterogeneity of one-carbon metabolism can be in part deduced from the patterns of gene expression across human tumors [8]. These gene expression profiles can constraint metabolic models to get predictions of metabolic fluxes [9]. These models are however imprecise because there are different layers of regulation between gene expression and reaction rates. Different experimental techniques have been used to quantify one-carbon metabolism, including metabolomics [10,11], ^3^H tracing [4,10,12,13], and reporter assays [5]. Yet, we still lack a systematic methodology for a comprehensive quantification of one-carbon metabolism fluxes.

There are established approaches to estimate metabolic fluxes, including metabolite consumption release (CORE) profiles [14], metabolic flux analysis (MFA) [15,16], and kinetic flux profiling (KFP) [17]. CORE profiles are used to estimate the exchange of metabolites between cells and the culture media. On top of CORE profiles, MFA exploits the relationship between metabolic fluxes and isotope fractions at steady state. However, not all metabolic fluxes can be inferred from the CORE profiles and the steady state isotope fractions. KFP allows for the estimation of additional fluxes knowing that the time scale for the transient dynamics of each metabolite is determined by the ratio between the metabolite concentration and its net flux of production/consumption. However, KFP requires measurement of isotope fractions at several time points, and therefore, the estimation of additional fluxes comes at a higher cost. Here we introduce a mixed approach where the benefits of these methods are integrated.

## Methods

### tMFA

The basic equations of tMFA are derived and reported in the Additional file 1.

#### Cells and culture media

MCF7, MDA-MB-231, and MDA-MB-468 breast cancer cell lines were obtained from ATCC. Cells were maintained in RPMI-1640 medium containing 10% fetal bovine serum (FBS). RPMI-1640 medium containing 10% dialyzed FBS was used for seeding and performing all experiments unless otherwise noted. All cell culture medium and FBS were purchased from Invitrogen (Carlsbad, CA, USA).

### ^13^C tracing (experiment 1, transient data, and TEPP-46)

#### Cell culture

6 × 10^5^ MCF7 cells were plated into each well of six-well tissue culture plates using RPMI-1640 medium containing 10% dialyzed FBS. The next day, culture medium was removed and replaced with serine-free RPMI-1640 medium containing 10% dialyzed FBS and 0.268 mM [U-^13^C]-L-serine without any drug or with 100 nM methotrexate (MTX) or 100 μM TEPP-46. Plates were incubated for 4, 8, and 16 h (MTX-treated cells) or 8 h (TEPP-46-treated cells) and processed as described below.

#### Metabolite extraction

Immediately after the incubation period, adhered cells were rapidly washed three times with chilled phosphate-buffered saline (PBS). Intracellular metabolites were extracted, in triplicate, by adding a volume equivalent to 1 × 10^6^ cells/mL with extraction solution at 4°C (methanol, acetonitrile, and water 5:3:2) and incubating the plate for 5 min at 4°C. The intracellular extract was collected and mixed for 10 min at 4°C before being centrifuged at 16,100×*g* for 10 min at 4°C to precipitate the proteins. The supernatants were transferred into HPLC vials and stored at −80°C until liquid chromatography mass spectrometry (LC-MS) analysis.

#### LC-MS analysis

An Exactive Orbitrap mass spectrometer (Thermo Scientific, Waltham, MA, USA) was used together with a Thermo Scientific Accela HPLC system. The HPLC setup consisted of a ZIC-pHILIC column (SeQuant, 150 × 2.1 mm, 5 μm, Merck KGaA, Darmstadt, Germany), with a ZIC-pHILIC guard column (SeQuant, 20 × 2.1 mm) and an initial mobile phase of 20% 20 mM ammonium carbonate, pH 9.4, and 80% acetonitrile. Cell extracts (5 μL) were injected, and metabolites were separated over a 15-min mobile phase gradient, decreasing the acetonitrile content to 20%, at a flow rate of 200 μL/min and a column temperature of 45°C. The total analysis time was 23 min. All metabolites were detected across a mass range of 75 to 1,000 *m*/*z* using the Exactive mass spectrometer at a resolution of 25,000 (at 200 *m*/*z*), with electrospray ionization (ESI) and polarity switching to enable both positive and negative ions to be determined in the same run. Lock masses were used, and the mass accuracy obtained for all metabolites was below 5 ppm. Data were acquired with Thermo Xcalibur software (Thermo Scientific, Waltham, MA, USA).

#### Peak areas

The peak areas of different metabolites were determined using Thermo TraceFinder software (Thermo Scientific, Waltham, MA, USA) where metabolites were identified by the exact mass of the singly charged ion and by known retention time on the HPLC column. Commercial standards of all metabolites detected had been analyzed previously on this LC-MS system with the pHILIC column. The ^13^C-labeling patterns were determined by measuring peak areas for the accurate mass of each isotopologue of many metabolites. Intracellular metabolites were normalized to protein content of the cells, measured at the end of the experiment by the Lowry assay.

### ^13^C tracing (experiment 2, data across cell lines)

#### Cell culture

3 × 10^6^ (MCF7), 3 × 10^6^ (MDA-MB-468), or 4 × 10^6^ (MDA-MB-231) cells were plated into 10-cm plates using RPMI medium containing 10% dialyzed FBS. The next day, culture medium was removed and replaced with serine-free RPMI medium containing 10% dialyzed FBS and 0.268 mM U-^13^C-serine (MCF7 or MDA-MB-231, with or without 100 nM MTX) or 11.1 mM U-^13^C-glucose (MDA-MB-468, with or without 100 nM MTX; MDA-MB-231, with or without 2.5 μM atorvastatin). Plates were incubated for 8 h and then harvested and processed as described below.

#### Metabolite extraction

The culture medium was aspirated from the plates, and cells were washed twice by 5% mannitol solution (10 mL first and then 2 mL). The cells were then treated with 800 μL of methanol and left at rest for 30 s in order to inactivate enzymes. Next, the cell extract was treated with 550 μL of Milli-Q water containing internal standards (H3304-1002, Human Metabolome Technologies, Inc., Tsuruoka, Japan) and left at rest for another 30 s. The extract was obtained and centrifuged at 2,300×*g* and 4°C for 5 min, and then 800 μL of upper aqueous layer was centrifugally filtered through a Millipore 5-kDa cutoff filter at 9,100×*g* and 4°C for 120 min to remove proteins. The filtrate was centrifugally concentrated and re-suspended in 50 μL of Milli-Q water for capillary electrophoresis mass spectrometry (CE-MS) analysis.

#### CE-MS

CE-time-of-flight mass spectrometry (TOFMS) was carried out using an Agilent CE Capillary Electrophoresis system equipped with an Agilent 6210 Time of Flight mass spectrometer, Agilent 1100 isocratic HPLC pump, Agilent G1603A CE-MS adapter kit, and Agilent G1607A CE-ESI-MS sprayer kit (Agilent Technologies, Waldbronn, Germany). The systems were controlled by Agilent G2201AA ChemStation software version B.03.01 for CE (Agilent Technologies, Waldbronn, Germany). The metabolites were analyzed by using a fused silica capillary (50 μm *i.d.* × 80 cm total length), with commercial electrophoresis buffer (solution ID: H3301-1001 for cation analysis and H3302-1021 for anion analysis, Human Metabolome Technologies, Inc., Tsuruoka, Japan) as the electrolyte. The sample was injected at a pressure of 50 mbar for 10 s (approximately 10 nL) in cation analysis and 25 s (approximately 25 nL) in anion analysis. The spectrometer was scanned from *m/z* 50 to 1,000. Other conditions were as described previously [18-20].

#### Peak areas

Peaks were extracted using automatic integration software MasterHands (Keio University, Tsuruoka, Japan) in order to obtain peak information including *m/z*, migration time (MT) for CE-TOFMS measurement, and peak area. Signal peaks corresponding to adduct ions and other product ions of known metabolites were excluded, and the remaining peaks were annotated with putative metabolites and their isotopic ions from the HMT metabolite database based on their MTs and *m*/*z* values determined by TOFMS. The tolerance range for the peak annotation was configured at ±0.5 min for MT and ±30 ppm for *m/z*. In addition, peak areas were normalized against those of the internal standards, and then the resultant relative area values were further normalized by sample amount.

#### TEPP-46 growth inhibition

5 × 10^3^ cells per well were plated in 96-well plates in Roswell Park Memorial Institute medium (RPMI)-1640 media containing 10% dialyzed FBS. The next day, medium was removed and replaced with fresh medium containing varying concentrations of MTX with and without 100 μM TEPP-46. Plates were incubated for 72 h. Cell viability was assayed using the Cell Titer 96 Aqueous One Solution (MTS) assay, performed according to the manufacturers’ protocol (Promega, Madison, WI, USA).

#### Protein and phosphoprotein expression

Twenty-four hours before methotrexate treatment, 3 × 10^6^ MCF7, MDA-MB-468, or MDA-MB-231 cells were seeded in 10-cm plates in RPMI medium supplemented with 10% dialyzed FBS and incubated overnight at 37°C in an incubator with 5% CO_2_. At the time of treatment, culture medium was removed and replaced with fresh medium or fresh medium containing 100 nM MTX and placed into a 37°C incubator with 5% CO2. At the sampling time points, culture medium was removed and cells were rinsed with PBS (Gibco, Grand Island, NY, USA) and scraped into microcentrifuge tubes. After brief centrifugation, cell pellets were lysed in RIPA buffer containing a commercial protease inhibitor mix (Roche, Nutley, NJ, USA) and phosphatase inhibitor (50 mM sodium fluoride, 10 mM sodium orthovanadate). After protein quantification by the Bradford protein assay (Bio-Rad Laboratories, Hercules, CA, USA), proteins were resolved by 10% SDS-PAGE and transferred onto a nitrocellulose membrane (Bio-Rad Laboratories, Hercules, CA, USA). After blocking the membrane with 5% nonfat dry milk prepared in tris-buffered saline + 0.1% Tween-20, the membrane was incubated with the desired primary antibody according to the manufacturer’s directions at 4°C overnight. The membrane was washed in tris-buffered saline + 0.1% Tween-20 and incubated for 2 h at room temperature with the appropriate peroxidase-conjugated secondary antibody. Bands were visualized using an enhanced chemiluminescence kit (Pierce, Thermo Fisher Scientific, Rockford, IL, USA). Anti-AMP-activated protein kinase (AMPK), anti-Thr172 pAMPK, anti-acetyl-CoA-carboxylase (ACC), anti-Ser79 ACC, and anti-rabbit secondary were purchased from Cell Signaling Technology (Danvers, MA, USA). Anti-glyceraldehyde 3-phosphate dehydrogenase (GAPDH) was purchased from Millipore (Billerica, MA, USA), and anti-mouse secondary was purchased from Santa Cruz Biotechnologies (Dallas, TX, USA).

#### Metabolic flux estimation errors

The errors in the flux estimations could be only due to the fitting of the tMFA model equations for purines (Additional file 1, equations 2.1.1 and 2.1.2) to the observed data. In all conditions analyzed, we obtained chi-squared test *P* values for the fitting that are orders of magnitude below 0.05. Beyond that, the number of parameters equals the number of measurements required to solve the tMFA equations. There are no significant fitting errors compared to the variations we observe between replicates.

#### Statistical analyses

**Figure 2:**
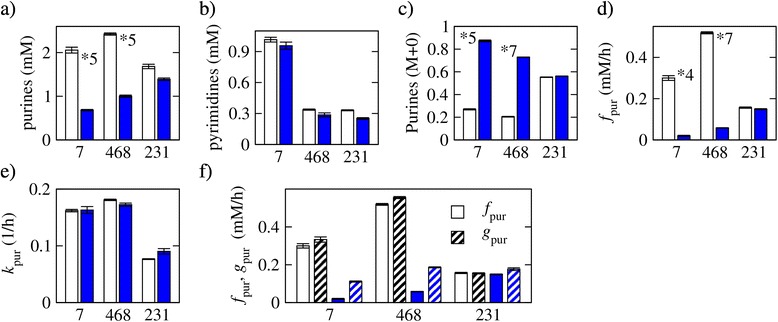
Inhibition of purine synthesis. **(a)** Purine concentration (*C*_pur_) 8 h after plating in untreated (open bars) and MTX-treated (filled bars) cells: MCF7 (7), MDA-MB-468 (468), and MDA-MB-231 (231). **(b)** Pyrimidine concentrations at the same time point. **(c)** Fraction of unlabeled purines. **(d)** Estimated purine synthesis rate. **(e)** Estimated purine turnover rate per unit of purines. **(f)** Balance between the purine synthesis (*f*_pur_) and consumption (*g*_pur_ = *k*_pur_ *C*_pur_) rates. The white and blue bars represent the rate of purine synthesis (white, untreated; blue, MTX-treated), and the dashed black and dashed blue bars represent the rate of purines consumption (black, untreated; blue, MTX-treated). The symbol *X denotes a difference between untreated and MTX-treated with a statistical significance less than 5 × 10^−*X*^.

**Figure 3:**
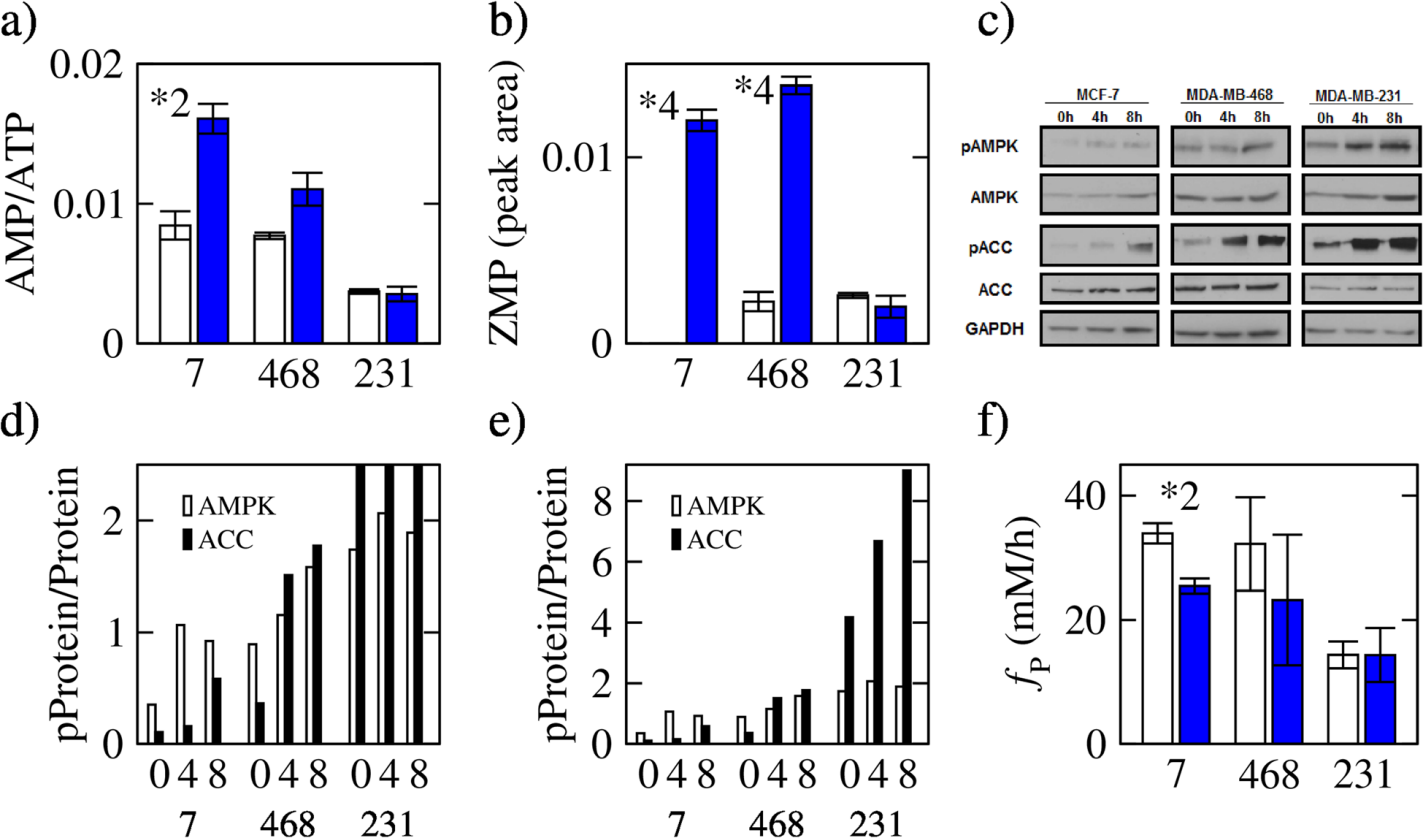
Energy stress and inhibition of protein synthesis. **(a)** AMP to ATP ratio in untreated cells (open white bars) and cells treated with MTX (filled blue bars). **(b)** ZMP levels. Not detected in untreated MCF7 cells. **(c)** Protein and phosphoprotein levels at different times after MTX treatment. **(d**,**e)** Ratio of phosphorylated to total protein levels using different scales for the *Y*-axis. **(f)** Protein synthesis rate estimated from the uptake of essential amino acids. Bars represent the average and error bars the standard deviation over three replicates. The symbol *X denotes a difference between untreated and MTX-treated with a statistical significance less than 5 × 10^−*X*^.

**Figure 4:**
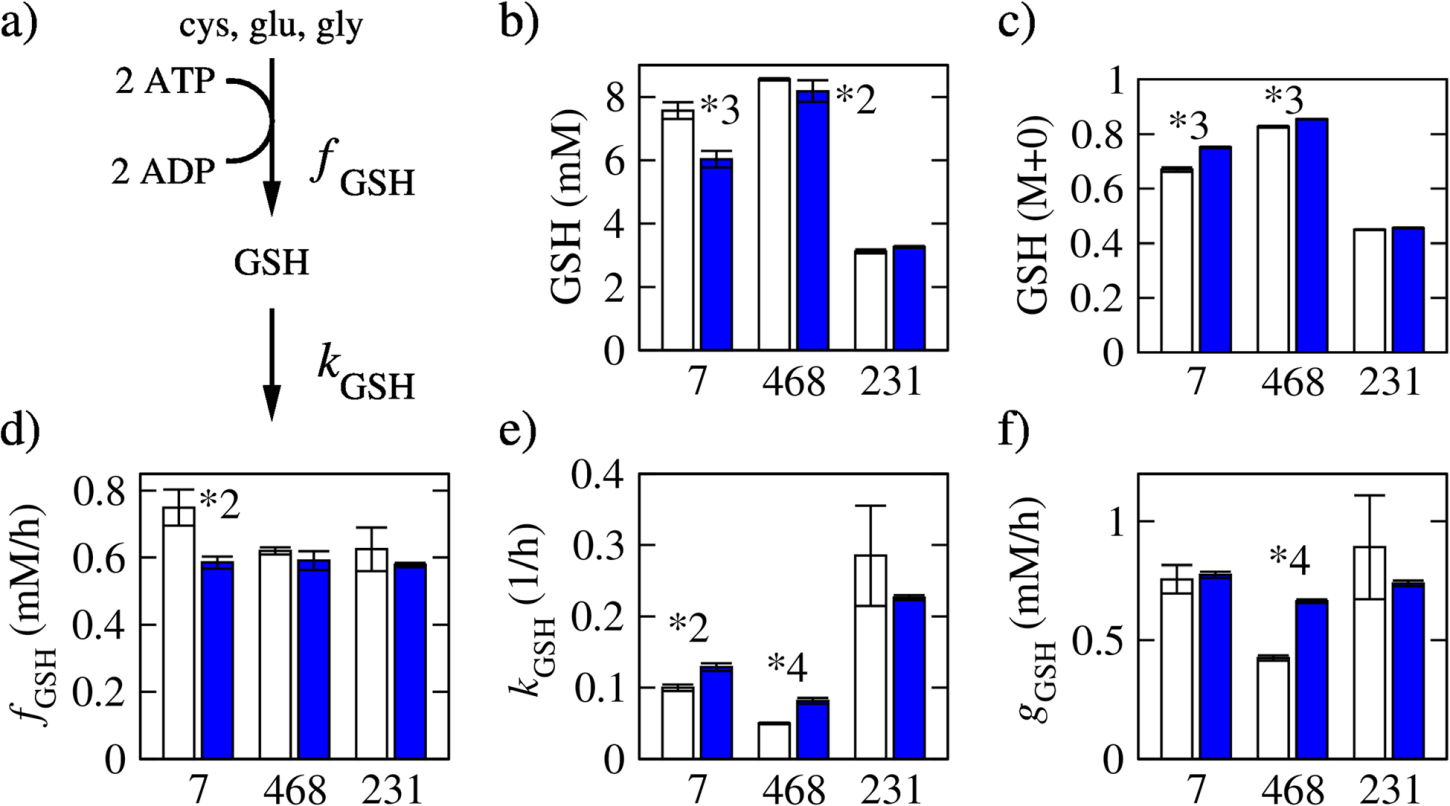
Glutathione balance. **(a)** GSH synthesis and turnover. **(b)** GSH concentration (*C*_GSH_) 8 h after plating in untreated (open bars) and MTX-treated (filled bars) cells. **(c)** Fraction of unlabeled GSH. **(d)** Estimated GSH synthesis rate. **(e)** Estimated GSH turnover rate. **(f)** Estimated GSH consumption rate (*g*_GSH_ = *k*_GSH_ *C*_GSH_). Bars represent the average and error bars the standard deviation over three replicates. The symbol *X denotes a difference between untreated and MTX-treated with a statistical significance less than 5 × 10^−*X*^.

**Figure 5:**
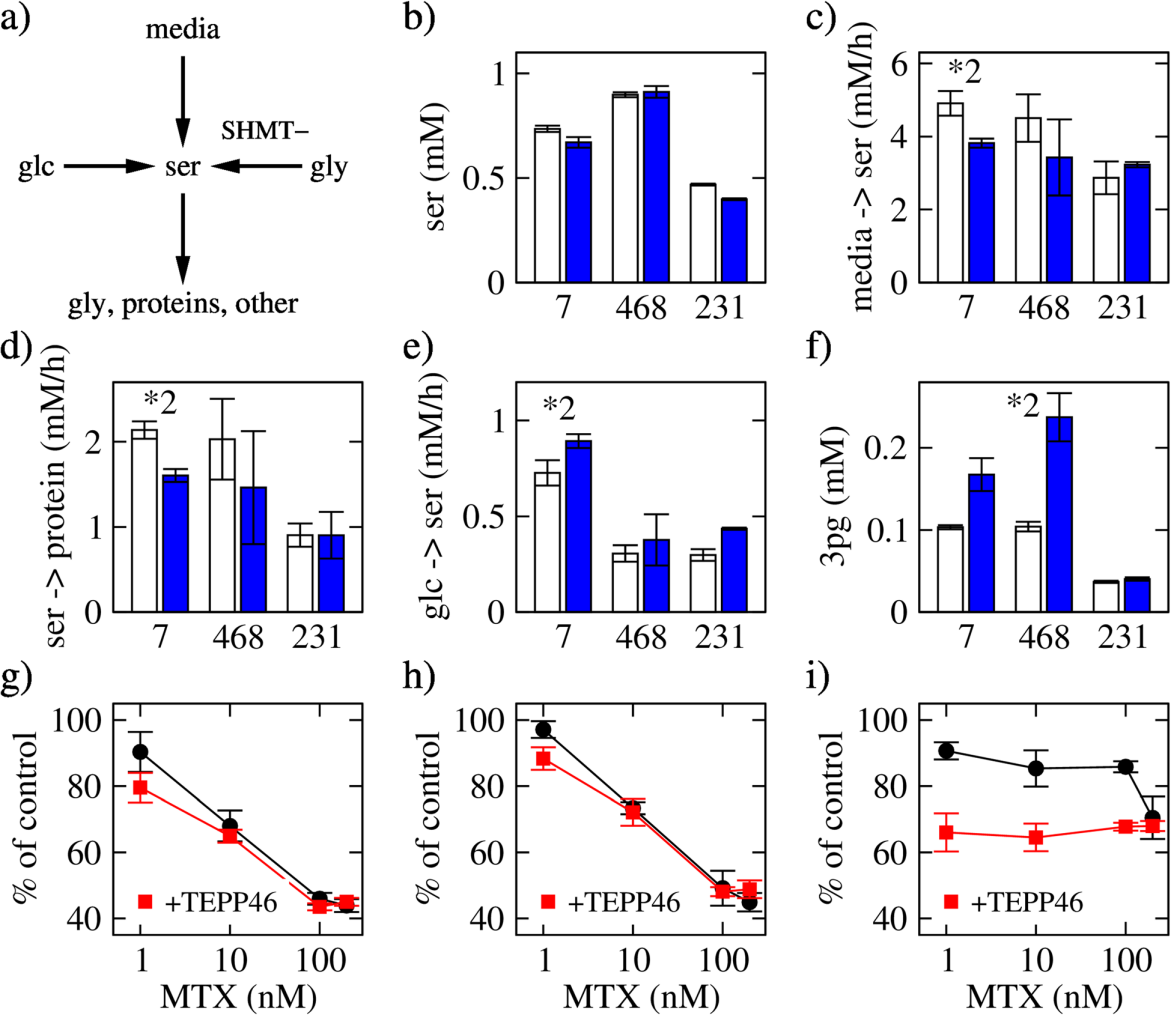
Serine balance. **(a)** Serine synthesis and consumption. Abbreviations: glc, glucose; ser, serine; gly, glycine; SHMT-, reverse SHMT. **(b)** Serine concentration 8 h after plating in untreated (open bars) and MTX-treated (filled bars) cells. **(c)** Serine uptake rate. **(d)** Serine consumption by protein synthesis. **(e)** Serine synthesis rate from glucose. **(f)** 3-phosphoglycerate (3PG) concentration. **(g**-**i)** Growth inhibition by co-treatment with MTX (1 to 100 nM) and the PKM2 activator TEPP-46 (100 μM). Bars represent the average and error bars the standard deviation over three replicates. The symbol *X denotes a difference between untreated and MTX-treated with a statistical significance less than 5 × 10^−*X*^.

**Figure 6:**
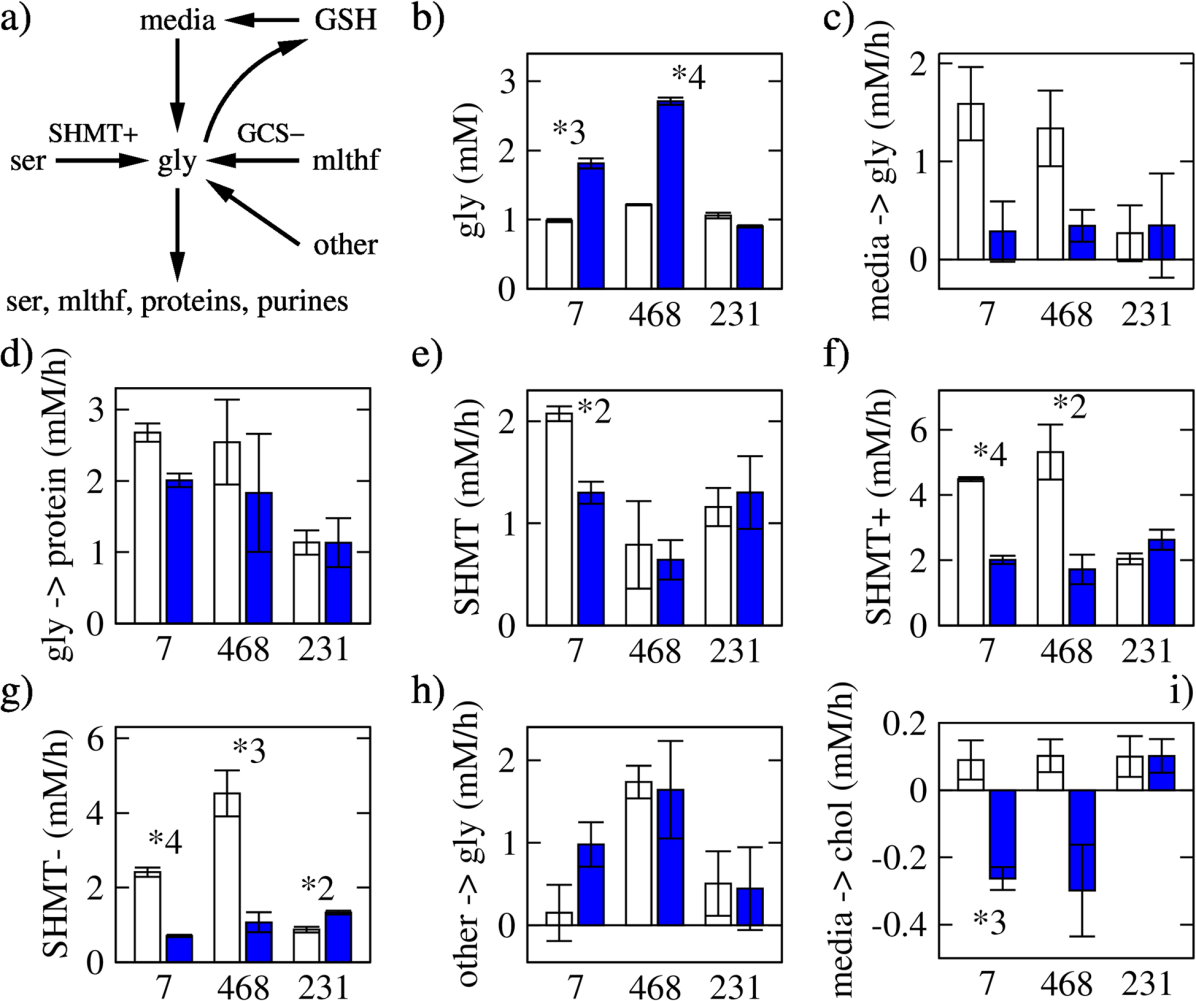
Glycine accumulation. **(a)** Glycine synthesis and consumption. Abbreviations: SHMT+, forward SHMT; GCS-, reverse GCS; mlthf, 5,10-methylene-tetrahydrofolate. **(b)** Glycine concentration 8 h after plating in untreated (open bars) and MTX-treated (filled bars) cells. **(c)** Estimated glycine exchange rate after corrected for GSH turnover. Positive values indicate uptake, and negative values release. **(d)** Glycine consumption for protein synthesis. **(e)** Net SHMT rate (forward minus backward). **(f)** Forward SHMT rate. **(g)** Backward SHMT rate. **(h)** Glycine production from unknown sources. **(i)** Exchange rate of choline. Positive values indicate uptake and negative values release. Bars represent the average and error bars the standard deviation over three replicates. The symbol *X denotes a difference between untreated and MTX-treated with a statistical significance less than 5 × 10^−*X*^.

**Figure 7:**
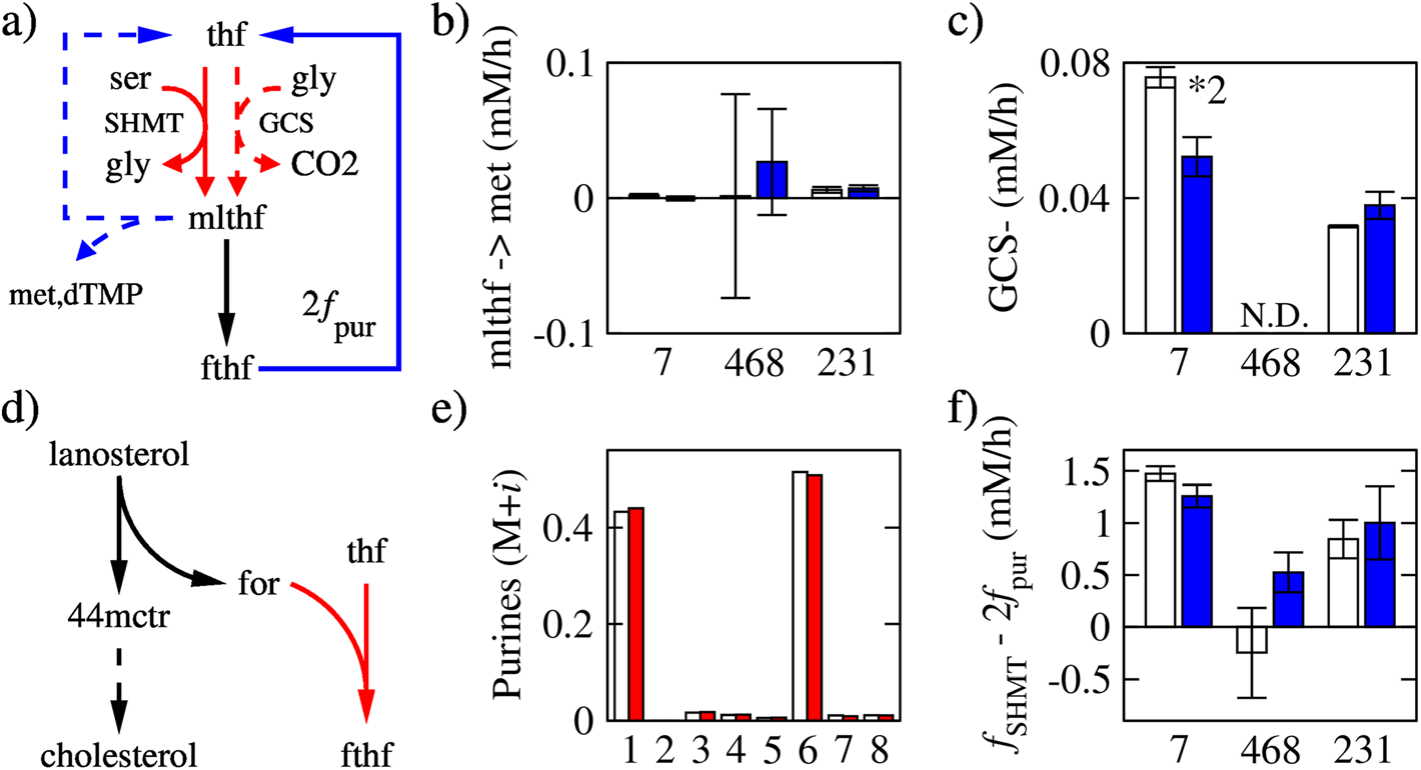
THF balance. **(a)** Reactions where a one-carbon unit is incorporated into (red) or released from (blue) folates. Abbreviations: thf, tetrahydrofolate; mlthf, 5,10-methylene-thf; fthf, 10-formyl-thf; met, methionine; dTMP, thymidylate. **(b)** Rate of one-carbon transfer from mlthf to methionine. **(c)** Backward rate of glycine cleavage. It could not be estimated in MDA-MB-468 because the glycine M+1 fraction was close to the detection limit. **(d)** Formate released by the cholesterol synthesis pathway. Abbreviations: 44mctr, 4,4-dimethyl-5-α-cholesta-8,14,24-trien-3β-ol; for, formate. **(e)** Purine isotope fractions in untreated (white boxes) and atorvastatin-treated (red boxes) MDA-MB-231 cells. **(f)** Difference between one-carbon production by SHMT and one-carbon consumption by purine synthesis. Bars represent the average and error bars the standard deviation over three replicates. The symbol *X denotes a difference between untreated and MTX-treated with a statistical significance less than 5 × 10^−*X*^.

All the metabolic flux estimations were carried out using input data for one technical replicate at the time. The metabolite concentrations, isotope fractions, and estimated fluxes reported in Figures 1, 2, 3, 4, 5, 6, and 7 represent the average and standard deviation (error bars) across three technical replicates. All reported *P* values were determined by comparing the three technical replicates from untreated and three technical replicates from treated cells using ANOVA.

## Results and discussion

### Transient metabolic flux analysis

After the addition of a tracer to a cell culture, an intracellular metabolite pool can be divided into unlabeled metabolite remaining from the initial pool (residual) and unlabeled/labeled metabolite synthesized during the course of the experiment (Figure 1b). We can chose to interrogate metabolites at an intermediate time where some metabolites are at steady state (no residual) and others are still following their transient dynamics. In this context, we can use a hybrid approach between MFA and KFP to infer the metabolic fluxes (Additional file 1). The resulting mathematical equations have a structure similar to those derived in MFA [15], except for a parameter *ε* quantifying the transient amount of residual. Thus, we name this methodology transient MFA (tMFA). tMFA can be used to investigate the effect of interventions on metabolism (Figure 1c). By comparing the estimated synthesis rate between untreated and treated conditions, we can quantify the inhibition of metabolite synthesis.

The experimental protocol implementing tMFA is sketched in Figure 1d. Media samples are taken at plating (*t* = 0) and at sampling (*t* = *T*) times to estimate exchange rates between the media and cells. Cell samples are taken at the sampling time to quantify intracellular metabolite concentrations and isotope fractions. The quantification of media and cell metabolite concentrations and isotope fractions can be done using metabolomic techniques. The tMFA equations are then used to infer the metabolic fluxes from these measurements. On its current implementation, tMFA is based on two major assumptions (i) that the isotope fractions of steady state metabolites are approximately constant in time and (ii) that the turnover rate of transient metabolites is approximately constant in time.

Here we use tMFA to quantify folate metabolism fluxes (Figure 1a) and their response to treatment with the antifolate MTX, a well-known inhibitor of purine synthesis [12]. To interrogate folate metabolism using tMFA, we first validated the tMFA approximations. In a first experiment (Exp 1), we performed [U-^13^C]-L-serine tracing in MCF7 cells at different time points (4, 8, and 16 h after addition of the tracer) and quantified the ^13^C fractions using LC-MS. When treating cells with MTX, the drug was added at the 0-h time point. In a second experiment (Exp 2), we performed [U-^13^C]-L-serine tracing in MCF7 cells at 8 h after addition of the tracer and quantified the ^13^C fractions using CE-MS. To take into account the potential variations in cell size, we normalized the metabolite concentrations and fluxes by the cell volume. All concentrations are reported in mol/L cell (molar, M) and the fluxes in mol/h/L cell (M/h).

Intracellular serine has a concentration (*C*) in the mM range and the net flux of serine production/consumption (*f*) is in the mM/h range, resulting in a typical transient time scale (*τ~C/f*) of about 1 h. Therefore, serine could be treated as a steady state metabolite at the 8-h sampling point. The measured intracellular ^13^C fractions are indeed almost constant in time from 4 to 16 h in both untreated (Figure 1e) and MTX-treated (Figure 1f) MCF7 cells. There is a small but appreciable decay of the M+3 serine fraction. This is most likely due to a leaking of serine from cells to the media, in spite of a net serine uptake. This leaking can be deduced from the measurement of an about 10% M+0 fraction of serine in the culture media at 8 h, which originally contained only M+3 serine. This unlabeled serine comes from *de novo* serine synthesis from glucose. The unlabeled serine pool will increase in time while M+3 serine pool is fixed to the amount originally in the media. Therefore, [U-^13^C]-L-serine tracing experiments beyond 8 h will be forced to take this leaking into consideration.

Similar to serine, intracellular glycine has a concentration in the mM range and the net flux of glycine production/consumption is in the mM/h range, resulting on a typical transient time scale of about 1 h. Therefore, glycine could be also treated as a steady state metabolite at the 8-h sampling point. The measured ^13^C glycine fractions are at steady state between 8 and 16 h in both untreated (Figure 1g) and MTX-treated MCF7 cells (Figure 1h).

The purine intracellular pool has also a concentration in the mM range, but the net flux of purine production/consumption is in the 0.1-mM range (see estimates below), resulting on a typical transient time scale of about 10 h. Therefore, purines should be treated as transient metabolites when profiling cells around 8 h. In the current implementation of tMFA, we assume that transient metabolites have a constant turnover rate per unit of metabolite (*k*), defined as the net rate of metabolite consumption divided by the metabolite concentration. The turnover rate of purines (*k*_pur_) at a given time point can be estimated from the purine ^13^C fractions measured at that time point (Additional file 1). In untreated MCF7 cells, the estimated *k*_pur_ is approximately constant between 4 and 8 h but drops at 16 h (Figure 1i). In MCF7 cells treated with MTX *k*_pur_ decays gradually (Figure 1j), but the difference between the value at 4 and 8 h is small and comparable to the variation between the two independent experiments. Based on this data, we conclude that *k*_pur_ is approximately constant between 4 and 8 h and we can apply tMFA to purines within this time window.

The concentration of glutathione (GSH) is around 6 mM while its production/consumption rate is 0.6 mM/h (as reported below), resulting on a typical transient time scale of about 10 h. Therefore, glutathione should also be treated as transient metabolite when profiling cells around 8 h, and we should validate the tMFA assumption of a constant glutathione turnover rate (*k*_GSH_). *k*_GSH_ can be estimated at a given time point from the GSH-labeling fractions at that time point. We observed that the estimated *k*_GSH_ is approximately constant between 4 and 16 h in untreated (Figure 1k) and MTX-treated (Figure 1l) MCF7 cells. Based on this data, we conclude that *k*_GSH_ is approximately constant between 4 and 16 h and we can apply tMFA to glutathione within this time window.

The analysis of the transient ^13^C data indicates 8 h as an optimal choice to apply tMFA. At earlier times, the ^13^C fractions of glycine are still following a transient dynamics. At later times, the assumption of a constant purine turnover rate does not hold and the serine ^13^C fractions depart from steady state. To further validate the choice of 8 h as an optimal sampling point, we estimated the purine and glutathione turnover rates using the 8-h data generated in Exp 2. Then we made predictions for the time profiles of the purine and glutathione unlabeled fractions, assuming a constant turnover rate equals to the estimated value. In the case of purines, the predicted time profiles are in good agreement with those measured in Exp 1 at 4 and 8 h in both untreated and treated cells (Figure 1m,n). However, deviations are observed at the 16-h time point. In the case of glutathione, the predicted time profiles are in good agreement with those measured in Exp 1 at all the time points collected in both untreated and treated cells (Figure 1o,p). This validation not only supports the use of 8 h as an optimal sampling point but also demonstrates the stability of the estimates across independent experiments. Furthermore, since the samples collected in Exp 1 and Exp 2 were analyzed with LC-MS and CE-MS, respectively, this validation also demonstrates the stability of our estimates across MS platforms. Finally, we profiled MDA-MB-231 cells using two different tracers, [U-^13^C]-L-serine and [U-^13^C]-D-glucose, allowing us to compare the reliability of tMFA estimates across tracers. The turnover rate estimations for purines and glutathione using either tracer and in good agreement, demonstrating that tMFA is stable across the tracers (Additional file 1: Figure S1).

In the following, we analyze folate metabolism fluxes using targeted metabolomics and ^13^C tracing in MCF7 and other breast cancer cell lines. Based on the analysis above, we profiled cells 8 h after addition of the ^13^C tracer. The cell lines MCF7, MDA-MB-468, and MDA-MB-231 were selected to represent high (IC50 = 5 nM), medium (60 nM), and low (1,000 nM) sensitivity to MTX treatment, respectively. We chose a MTX concentration of 100 nM, above the IC50 for the most sensitive cell line MCF7 but below the IC50 for the most insensitive cell line MDA-MB-231. Unless otherwise specified, the MCF7 and MDA-231 cell lines were profiled using [U-^13^C]-L-serine and the MDA-MB-468 cell line using [U-^13^C]-D-glucose as the tracer, in both cases resulting in partial labeling of the metabolites of interest. We choose [U-^13^C]-D-glucose for the MDA-MB-468 cell line based on a previous report indicating that these cell lines do not uptake serine [3], although these cells did uptake serine in our culture conditions. All concentrations are reported in mol/L cell (molar, M) and the fluxes in mol/h/L cell (M/h).

### Inhibition of purine synthesis

Upon treatment with MTX, we observed a twofold decrease of the purine concentration in MCF7 and MDA-MB-468 cells but to a lesser extent in MDA-MB-231 cells (Figure 2a). As a control, the concentration of pyrimidines remains practically unchanged in the three cell lines (Figure 2b). We also observed a fourfold increase in the unlabeled purine fraction in MCF7 and MDA-MB-468 cells (Figure 2c), which could be the outcome of inhibition of purine synthesis from labeled precursors. Modeling purines as a transient metabolite, we estimated their synthesis and turnover rates. There is about a tenfold decrease in the rate of purine synthesis in MCF7 (*P* = 1.4 × 10^−4^) and MDA-MB-468 (*P* < 1.5 × 10^−7^) but just a slight (less than twofold, *P* = 0.20) decrease for MDA-MB-231 (Figure 2d). In the MCF7 cells, the purine turnover per unit of purine (*k*_pur_) is not affected by MTX treatment and there is just a slight increase in the MDA-MB-468 and MDA-MB-231 cells (Figure 2e). Taken together, these data indicate that the reduction of the purine concentration at 8 h is the result of inhibition of purine synthesis with no change in the purine turnover rate.

In untreated cells, we would expect a steady state where the purine production rate (*f*_pur_) is balanced by the purine consumption rate (*g*_pur_), the latter being equal to the purine turnover per unit of purine times the purine concentration (*g*_pur_ = *k*_pur_*C*_pur_). Indeed, in untreated cells, the tMFA estimates of *f*_pur_ and *g*_pur_ are approximately equal independently of the cell line (Figure 2f). In contrast, the purine consumption rate exceeds its production rate in the MCF7 and MDA-MB-468 cells following MTX treatment (Figure 2f), consistent with the drop in the purine concentration (Figure 2a).

### Energy stress

Although the concentration of purine nucleotides decreases following MTX treatment (Figure 2a and Additional file 1: Figure S2), the changes are relatively different for each nucleotide. We observe a significant increase in the AMP/ATP ratio in the MCF7 and MDA-MB-468 cells (Figure 3a), while the ADP/ATP ratio remains approximately constant (Additional file 1: Figure S2d). We also observe a significant increase in the ZMP levels in MCF7 and MDA-MB-468 cells (Figure 3b). Increases of both the AMP/ATP ratio and ZMP are signals that stimulate the AMP kinase (AMPK) [21]. Indeed, following MTX treatment, we observe an increase in the phosphorylation of AMPK (pAMPK) relative to the AMK level in the MTX sensitive MCF7 and MDA-MB-231 cell lines but not in MDA-MB-231 (Figure 3c,d). In the MCF7 and MDA-MB-468 cell lines, the increase in the pAMPK/AMPK ratio is followed by an increase in the phosphorylation of the AMPK target acetyl CoA carboxylase (ACC) (Figure 3c,d). In the MDA-MB-231 cell line, there is also an increase in ACC phosphorylation (Figure 3e). However, since there is no trend in the pAMPK/AMPK ratio, we conclude that in MDA-MB-231 cells, the increased ACC phosphorylation is due to an AMPK-independent mechanism. We also notice that the level of phosphorylation of both AMPK and ACC is overall higher in MDA-MB-231 relative to MCF7 and MDA-MB-468, but this difference is observed already in the absence of MTX treatment (Figure 3e, 0-h time point). Thus, MTX treatment causes energy stress only in the MTX sensitive cell lines MCF7 and MDA-MB-468.

Protein synthesis requires a high rate of ATP turnover, and therefore, we hypothesized that MTX treatment should indirectly inhibit protein synthesis. To estimate the rate of protein synthesis, we used the uptake rate of essential amino acids as a surrogate [22]. We have previously shown that the protein synthesis rate estimated by this approach is proportional to the protein synthesis rate estimated from the rate of ^3^H-leucine incorporation into protein [22]. Following MTX treatment, there is a significant decrease in the protein synthesis rate in MCF7 cells (*P* = 0.03, Figure 3f), a similar trend in MDA-MB-468 cells (*P* = 0.63, Figure 3f), and no change in MDA-MB-231 cells (*P* = 0.68, Figure 3f).

The synthesis of GSH is energy dependent (Figure 4a), and it could also be inhibited by the energy stress caused by MTX treatment. Upon MTX treatment, the GSH concentration decreases significantly in the two sensitive cell lines (*P* = 0.004, MCF7; *P* = 0.035, MDA-MB-468) (Figure 4b). The fraction of unlabeled GSH increases significantly in MCF7 (*P* = 0.0009) and MDA-MB-468 (*P* = 0.005) cells treated with MTX, but not in MDA-MB-231 cells (*P* = 0.12) (Figure 4c). The increase in the GSH unlabeled fraction may result from the inhibition of GSH synthesis form labeled precursors. Modeling GSH as a transient metabolite, we estimated the GSH synthesis and turnover rates. Following MTX treatment, there is a significant decrease in the glutathione synthesis rate in MCF7 (*P* = 0.022) but no significant change in MDA-MB-468 (*P* = 0.55) or MDA-MB-231 (*P* = 0.67) cells (Figure 4d). The glutathione turnover rate increases in the MCF7 (*P* = 0.024) and MDA-MB-468 (*P* = 0.00046) cells treated with MTX, but it does not change significantly in MDA-MB-231 (*P* = 0.49) cells (Figure 4e). However, in MCF7 cells, the increase in glutathione turnover (measured per unit of glutathione) is balanced by a decrease in the glutathione concentration (Figure 4b), resulting in no significant change in the net glutathione consumption rate (*g*_GSH_ = *k*_GSH_*C*_GSH_, Figure 4f). In contrast, in MDA-MB-468 cells, where the glutathione concentration does not change (Figure 4b), the decrease in turnover rate results in a significant increase (*P* = 0.00046) in the net glutathione consumption rate (Figure 4f).

Taken together, these data indicate that MTX treatment causes energy stress and inhibition of protein and glutathione synthesis, in a degree proportional to the growth inhibition sensitivity to MTX.

### Induction of serine synthesis

Serine is a source of one-carbon units, and its balance may be perturbed by MTX treatment. Serine can be imported from the media, synthesized from glucose, or derived from glycine via the reverse activity of SHMT (Figure 5a). There is no significant difference in the serine concentration of treated relative to untreated cells for any of the three cell lines (Figure 5b). The uptake of serine from the media decreases upon MTX treatment (Figure 5c) in a degree proportional to the decrease in the serine consumption rate for protein synthesis (Figure 5d). In contrast, we observe a significant increase of serine synthesis from glucose in MCF7 cells treated with MTX (*P* = 0.03), but not a significant increase in MDA-MB-468 (*P* = 0.78) or MDA-MB-231 (*P* = 0.064) cells (Figure 5e). The induction of serine synthesis is consistent with an increase of the 3-phosphoglycerate (3PG) concentration (*P* = 0.1, MCF7; *P* = 0.037; MDA-MB-468; *P* = 0.028, MDA-MB-231; Figure 5f), the branching point from glycolysis to serine synthesis.

To investigate the contribution of serine synthesis induction to growth inhibition by MTX, we modulated the activity of PKM2. PKM2 is an isoform of pyruvate kinase that is often overexpressed in cancer cells [23]. PKM2 can be found in an active tetramer or in an inactive dimer form. This feature allows cells to favor either pyruvate production (active tetramer) or production of precursor metabolites such as 3PG (inactive dimer). Using an allosteric activator of PKM2 that favors the tetramer conformation (TEPP-46) [24], we investigated if favoring production of pyruvate instead of 3PG would have an effect on the response to MTX. Treatment of MCF7 cells with 100 μM TEPP-46 resulted in a decrease in the serine levels and an increase in the pyruvate levels (Additional file 1: Figure S6), indicating that indeed TEPP-46 activates glycolysis and inhibits serine synthesis. However, there is no significant change in the growth inhibitory activity of MTX upon co-treatment with the PKM2 activator (Figure 5g,h,i). For MDA-MB-231 cells, we observe a significant growth inhibition when adding TEPP-46, but this effect is independent of the MTX concentration (Figure 5i). These data indicate that MTX treatment can induce the synthesis of serine from glucose, but this change does not contribute to the growth inhibitory activity of MTX.

### Accumulation of glycine

The inhibition of purine synthesis can perturb the balance of the purine precursor glycine. Glycine can be imported from the media and derived from serine via the SHMT forward activity (SHMT+), from GSH turnover, and other unaccounted sources (Figure 6a). The intracellular glycine concentration increases about twofold in MCF7 and MDA-MB-468 cells treated with MTX (Figure 6b). After correcting for the GSH turnover, we observe a net glycine uptake from the media for the three cell lines (Figure 6c). Similar to the serine uptake rate, the glycine uptake rate decreases upon MTX treatment in a degree proportional to the decrease in the glycine consumption rate for protein synthesis (Figure 6d). The net rate of serine to glycine conversion decreases significantly in MCF7 cells (*P* = 0.0091), but it does not change significantly for MDA-MB-468 (*P* = 0.86) and MDA-MB-231 (*P* = 0.68) cells (Figure 6e). We note however that in both sensitive MCF7 and MDA-MB-468 cells, the forward and backward rate of serine to glycine conversion decreases (Figure 6f,g). This can be explained by a decrease in the concentration of both THF (favoring the forward reaction) and of 5,10-methenyl-THF (favoring the backward reaction) following MTX treatment [10].

We also estimate the existence of an unknown source of glycine with a variable production rate depending on the cell line and treatment (Figure 6h). The basal rate of glycine production from the unknown source is negligible in MCF7 cells but increases upon MTX treatment (Figure 6h). The other two cell lines have a significant level of glycine production from unknown sources, and this does not change significantly when treated with MTX. Glycine can be derived from choline, and therefore, choline is a candidate for the unknown source of glycine. In untreated MDA-MB-468 cells, the glycine production rate from the unknown source (Figure 6h) exceeds the rate of choline uptake (Figure 6i). After MTX treatment, choline is actually released to the media in MCF7 and MDA-MB-468 cells (Figure 6i). Therefore, choline does not account for glycine production from the unknown source in MCF7 and MDA-MB-468 cells. In MDA-MB-231 cells, glycine production rate from the unknown source is small (Figure 6h) and about the same magnitude of choline uptake rate (Figure 6i).

### Balance of one-carbon units

Next, we focused on the balance between reactions donating/accepting one-carbon units. In addition to purines, one-carbon units can be donated to methionine and thymidylate (Figure 7a). We applied FBA to estimate the transfer rate of one-carbon units to methionine. The obtained values are below 0.03 mM/h (Figure 7b), tenfold lower than the purine synthesis rate (Figure 2d). The rate of thymidylate synthesis can be estimated based on the cells doubling time, DNA content, and thymine abundance in DNA. We obtain a thymidylate synthesis rate of about 0.02 mM/h, again tenfold lower than the purine synthesis rate (Figure 2d). Therefore, methionine and thymidylate synthesis rates are not major consumers of one-carbon units relative to purine synthesis.

We have also investigated other sources of one-carbon units besides serine. Glycine can donate a one-carbon unit via the GCS (Figure 1a). Based on the M+1 isotope fraction of glycine, we estimate the reverse glycine cleavage rate to be in the 0.04 to 0.08 mM/h range (Figure 7c). Although we cannot exclude that the forward rate may over exceed the backward rate, the reversibility of the GCS together with the lack of an appreciable backward rate of glycine cleavage indicates that the GCS is not a major source of one-carbon units in these cells.

Cholesterol synthesis is another potential source of one-carbon units. In an intermediate step of this pathway, a one-carbon from lanosterol is released as formate (Figure 7d). We have shown that cell lines that are highly sensitive to atorvastatin (including MDA-MB-231) depend on *de novo* cholesterol synthesis for proliferation [25]. To sort out the potential contribution of one-carbon units by cholesterol synthesis, we analyzed [U-^13^C]-D-glucose tracing data from MDA-MB-231 cells, untreated or treated with the cholesterol synthesis inhibitor atorvastatin. We observe significant labeling of citrate as expected from labeling of AcCoA from [U-^13^C]-D-glucose (Additional file 1: Figure S7). The one-carbon released as formate comes from AcCoA, and it should have a significant M+1 fraction that would be reflected in purines. However, there is no significant change in the purine isotope fractions between atorvastatin treated and untreated cells (*P* = 0.33, Figure 7e). Therefore, the formate released by the cholesterol synthesis pathway is not a significant source of one-carbon units.

Finally, we compared the net SHMT rate (generating a one-carbon unit from serine) and the rate of purine synthesis (consuming 2 one-carbon units per purine). In MCF7 and MDA-MB-231 untreated and all treated cell lines, the SHMT rate exceeds the one-carbon requirements for purine synthesis (Figure 7f). In these cells, there are additional reactions releasing one-carbon units. One candidate is the reverse activity of FTHFS releasing the one-carbon as formate and producing ATP [6,7]. Another candidate is the activity of FTHFD releasing the one-carbon as CO_2_ and producing NADPH [4]. While our current implementation of tMFA does not allow us to resolve these two contributions, it shows that in MCF7 and MDA-MB-231 cells, there is an excess production of one-carbon units from serine that is not accounted for by biosynthetic processes. In contrast, in untreated MDA-MB-468 cells, the SHMT rate is about the rate of one-carbon requirements for purine synthesis (Figure 7f).

### Unaccounted contributions

**Figure 8:**
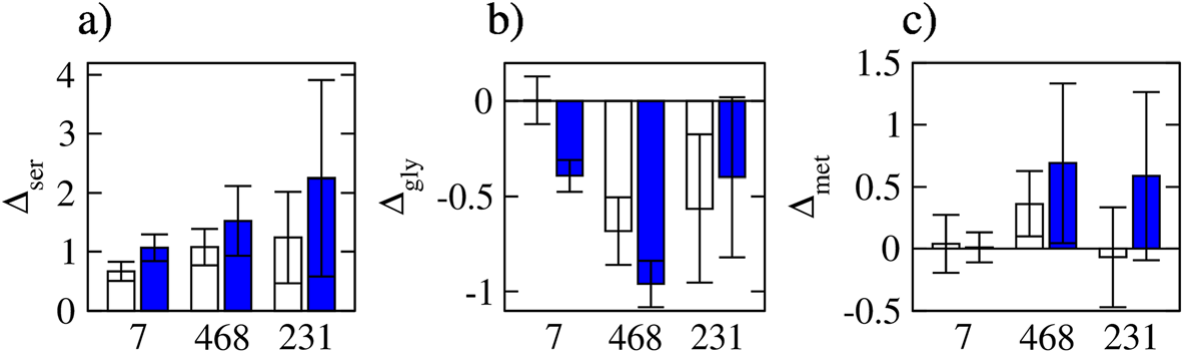
Balance of serine, glycine, and methionine: excess production (positive) or consumption (negative) flux for serine **(a)**, glycine **(b)**, and methionine **(c)** relative to their demand for protein synthesis, in untreated (open bars) and MTX-treated (filled bars) cells.

All metabolic flux analysis methodologies, including our tMFA implementation, are limited to the biochemical pathways build in into the calculations. To quantify contributions not accounted by the model, we made a balance of the production and consumption of each metabolite. As shown in the previous section, there is an excess production of one-carbon units above the requirements for purine synthesis. For serine, the uptake and synthesis from glucose exceeds the consumption rate from serine conversion to glycine and incorporation into protein, in an amount between one and two times the serine demand for protein synthesis (Figure 8a). Most likely, this excess production is consumed in the synthesis of cystathionine, phosphatidylserine, and sphinganine. However, the determination of those fluxes is beyond the focus of this work on folate metabolism. For glycine, we have the reported production from the unknown source (Figure 6h), which takes values between half and about the glycine demand for protein synthesis (Figure 8b). For methionine, we have shown above that there is no appreciable synthesis of methionine in the three breast cancer cell lines analyzed (Figure 7b). The methionine uptake approximately matches its demand for protein synthesis in MCF7 (untreated and MTX-treated) and MDA-MB-231 untreated cells (Figure 8c). In contrast, methionine is imported in excess of its demand for protein synthesis in MDA-MB-468 (untreated and MTX-treated) and MTX-treated MDA-MB-231 cells (Figure 8c), in an amount between 25% and 50% of the demand for protein synthesis. Most likely, the excess methionine uptake is consumed in the production of S-adenosylmethionine, which provides the methyl group for methylation. However, the determination of this flux is beyond the focus of this work on folate metabolism.

## Conclusions

tMFA is an extension of MFA incorporating transient isotope labeling data and taking advantage of a key feature of cell metabolism: the separation of time scales. By choosing to profile intracellular metabolites at an intermediate time point, we can divide metabolites into steady state and transient metabolites. In the context of folate metabolism, this includes the rate of purine and glutathione synthesis. Although tMFA takes advantage of transient data, it should not be confused with KFP [17]. The tMFA mathematical equations are quadratic on the fluxes and the transient parameter. The KFP equations are convolutions of exponential functions, they are more difficult to solve, and they require more input time points. On the other hand, more time points imply better estimates, and therefore, KFP remains the gold standard. We propose the use of tMFA in high-throughput applications and/or when the budget is limited.

Notably using tMFA, we demonstrate that MTX treatment results in a cascade of metabolic changes that are not simply a consequence of the reduction in the total purine pool. MTX also induces an increase in the AMP/ATP ratio, activation of the energy sensor AMPK, inhibition of energy consuming reactions such as protein and glutathione synthesis, and induction of serine synthesis. We also show that methionine and thymidylate syntheses are minor contributions to the balance of one-carbon units.

Pharmacological AMPK activation with the AICAR riboside has been reported to be synergistic with MTX treatment [26]. The proposed mechanism is accumulation of the AMP mimetic ZMP due to inhibition of purine synthesis by MTX. Indeed, MTX induces a significant increase in the ZMP levels in breast cancer cells sensitive to MTX. We also observe a significant increase in the AMP/ATP ratio in breast cancer cells sensitive to MTX treatment, in agreement with our previous report for a prostate cancer cell line [7]. We therefore propose that the increase of both the ZMP levels and the AMP/ATP ratio contributes to the AMPK activation due to MTX treatment.

The applications of tMFA go beyond what is presented above. The same methodology can be applied to quantify pyrimidine metabolism using a tracer that labels pyrimidine precursors. tMFA can be used to quantify the biosynthetic needs for glutamate and glutamine (proteins, purines, pyrimidines, glutathione, cysteine exchange). We therefore anticipate its use in the investigation of glutamine/glutamate metabolism. We also envision the application of tMFA to interrogate fatty acid metabolism, exploiting the transient time scale separation between AcCoA and fatty acids.

## Additional file

Additional file 1:
**Supplementary methods and supplementary figures.**

## Abbreviations

CE-MS: capillary electrophoresis mass spectrometry
CORE: consumption release profiles
dTMP: thymidylate
fthf: 10-formyl-thf
FTHFD: 10-formyl-tetrahydrofolate dehydrogenase
FTHFS: formyl-tetrahydrofolate synthase
GCS: glycine cleavage system
GCS-: reverse GCS
glc: glucose
gly: glycine
GSH: glutathione
KFP: kinetic flux profiling
LC-MS: liquid chromatography mass spectrometry
met: methionine
MFA: metabolic flux analysis
mlthf: 5,10-methylene-tetrahydrofolate
MTHFD: 5,10-methylene-tretrahydrofolate dehydrogenase
MTX: methotrexate
ser: serine
SHMT: serine hydroxymethyl transferase
SHMT−: reverse SHMT
SHMT+: forward SHMT
thf: tetrahydrofolate
tMFA: transient metabolic flux analysis
