# Supplementary material for: Quantification of folate metabolism using transient metabolic flux analysis
Source: Cancer Metab. 2015 May 28;3:6. doi: 10.1186/s40170-015-0132-6 (PMC4446824; doi:10.1186/s40170-015-0132-6)
Supplement: Additional file 1: — Supplementary methods and supplementary figures. [file 40170_2015_132_MOESM1_ESM.pdf]

Quantification of folate metabolism using transient metabolic flux analysis
Supplementary methods and figures

Philip M Tedeschi[1], Nadine Johnson-Farley[1], Hongxia Lin[1], Laura M Shelton[2], Takushi Ooga[3], Gillian Mackay[4], Niels Van Den Broek[4], Joseph R Bertino[1], Alexei Vazquez[1,4*]

[1] Rutgers Cancer Institute of New Jersey, New Brunswick, NJ 08901, USA

[2] Human Metabolome Technologies America, Boston, MA 02134, USA

[3] Human Metabolome Technologies, Tsuruoka, Yamagata 997-0052,Japan

[4] Cancer Research UK Beatson Institute, Glasgow, G20 8QW, UK

1- Transient metabolic flux analysis (tMFA)

Consider a cell culture in a medium containing one or more isotope labeled nutrients. Let us focus on an intracellular metabolite X with a time dependent total content $X(t)$ (mol/cell culture). The metabolite is divided in M+i isotope pools with amounts $X_i(t)$ ($\Sigma_i X_i = X$). The transient dynamics of the metabolite content and its isotope pools follow the equations

(1.1) $$\dot{X}(t) = N(t)\sum_j S_j f_j - N(t)g$$

(1.2) $$\dot{X}_i(t) = N(t)\sum_j \varphi_{ij} S_j f_j - N(t)g\frac{X_i(t)}{X(t)}$$

where $N(t)$ is the number of cells, S_j is the number of X molecules produced by reaction j, f_j is the rate of metabolite production from reaction j per cell, g is the net rate of metabolite consumption per cell, and φ_{ij} is the M+i isotope ratio of metabolite X when produced by reaction j. In equation (1.2) we observe that the negative term representing consumption of the M+i fraction depends on the ratio of g/X. By taking this fact into consideration we define the metabolite *turnover rate per unit of metabolite*

(1.3) $$k = \frac{N(t)g}{X(t)} = \frac{g}{n_X(t)}$$

where $n_X(t)=X(t)/N(t)$ is the amount of metabolite per cell. Using this definition we can rewrite equations (1.1) and (1.2) as

(1.4) $$\dot{X}(t) = N(t)\sum_j S_j f_j - kX(t)$$

(1.5) $$\dot{X}_i(t) = N(t)\sum_j \varphi_{ij} S_j f_j - kX_i(t)$$

When the metabolite content, turnover rate and consumption rate are in steady state, it is just a matter of choice whether we prefer to work with equations (1.1)-(1.2) or (1.4)-(1.5). However, when the system is in a transient state, one or the other system may apply depending of whether g or k is constant in time. Here we are interested in both unchallenged cells that could be in steady state or cells that have been challenged. In the latter case the metabolite concentration may decrease in time due to the inhibition of its synthesis. We assume that even in the transient context the rate of metabolite consumption can be approximated by first order kinetics, $g \approx kn(t)$, with a constant turnover rate per unit of metabolite k. This is an approximation and should be tested before the application of our framework. At the end we discuss the consequence of a time dependent turnover rate per unit of metabolite.

Assuming that the isotope fractions of all precursors required in the synthesis of X (implicitly included in φ_{ij}) and the turnover rate per unit of metabolite (k) are constant in time, from equations (1.4 we obtain

(1.6) $$X(t) = x(0)e^{-kt} + B(t)f_X^*$$

where

(1.7) $$f_X^* = \frac{1}{B(t)} e^{-kt} \int_0^t dt' N(t') e^{kt'} \sum_j S_j f_j(t')$$

is a weighted average across time of the net rate of X production per cell and

(1.8) $$B(t) = e^{-kt} \int_0^t dt' N(t') e^{kt'}$$

We notice that when the production rates are constant in time

(1.9) $$f_X^* = f_X = \sum_j S_j f_j$$

More generally, since the average in equation (1.7) is exponentially biased towards $f_j(t')$ values closer to the current time, we assume that

(1.10) $$f_X^*(t) \approx f_X(t) = \sum_j S_j f_j(t)$$

We define the parameter

(1.11) $$\varepsilon = \frac{X(0)e^{-kt}}{X(t)}$$

quantifying how close is metabolite X from achieving steady state ($\varepsilon << 1$). Using this definition, from equations (1.6) and (1.10) it follows that

(1.12) $$f_X \approx (1-\varepsilon)\frac{X(t)}{B(t)}$$

where the approximation sign follows from that in equation (1.10). Now let focus on the isotope fractions. Integrating equation (1.5) we obtain

(1.13) $$X_i(t) = \delta_{i0} X(0) e^{-kt} + B(t) \sum_j \varphi_{ij} S_j f_j^*$$

where δ_{i0}=1 if i=0 and δ_{i0}=0 otherwise, and

(1.14) $$f_j^*(t) = \frac{1}{B(t)} e^{-kt} \int_0^t dt' N(t') e^{kt'} f_j(t')$$

is a weighted average across time of the production rate f_j. Once again, since the average in equation (1.7) is exponentially biased towards $f_j(t')$ values closer to the current time, we assume that

(1.15) $$f_j^*(t) \approx f_j(t)$$

Dividing equation (1.13) by $X(t)$ and taking into account equations (1.11) , (1.12) and (1.15) we obtain

(1.16: tMFA) $$x_i = \delta_{i0}\varepsilon + (1-\varepsilon) \sum_j \varphi_{ij} S_j y_j$$

where $x_i = X_i(t)/X(t)$ is the isotope M+i fraction of X at time t and $y_j = f_j(t)/f_X(t)$. Equation (1.16) is the working expression of tMFA. We note that in the limit ε<<1 the tMFA equation (1.16) reduces to

(1.17: MFA) $$x_i \approx \sum_j \varphi_{ij} S_j y_j$$

which is essentially the MFA limit. Based on the similarity of equations (1.16) and (1.17) and the fact that ε quantifies the proximity to steady state we name equation (1.16) the transient MFA (tMFA) equation.

Solving tMFA

For a system with several metabolites we can divide the metabolites into steady state metabolites (ε<<1) and transient metabolites (ε~1). We will then write a MFA equation (1.17) for each steady state metabolite and a tMFA equation (1.16) for each transient metabolite. The tMFA sub-problem for transient metabolites is solved in three steps. First, if x_i, φ_{ij} and S_j are known we can calculate y_j and ε by solving the quadratic system of equations (1.17). If there are less equation than variables then the system does not have a unique solution and tMFA is not sufficient to calculate y_j and ε. If there are as many equations as variables then we obtain a unique value of y_j and ε. If there are more equations than variables we can calculate y_j and ε from the mean squared error solution of equation (1.17).

In the case where we obtain a unique solution for y_j and ε, we can calculate the turnover rate from equation (1.11)

(1.20) $$k = \frac{1}{t} \ln\left(\frac{X(0)}{X(t)\varepsilon}\right)$$

Then we can use equation (1.8) to calculate $B(t)$. Approximating the integral by the trapezoidal rule with n intervals of uniform length $\Delta t=t/n$ we obtain

$$(1.21) \qquad B(t) \approx \frac{\Delta t}{2} \sum_{i=1}^{n} \left[N(t_{i+1}) e^{-kt_{i+1}} + N(t_i) e^{-kt_i} \right]$$

Measuring $N(t)$ at the i=1,…,n time points and using the calculated value of k (1.20) we estimate $B(t)$ using equation (1.21). Once we have calculated $B(t)$ we can calculate f_X using equation (1.12) and then the fluxes $f_j=y_j f_X$.

Estimating ε in the context of exponential growth

In the context of exponential growth $N(t)=N_0\exp(\mu t)$, where μ is the growth rate. In this case from equations (1.8) and (1.12) we obtain

$$(1.22) \qquad \varepsilon = 1 - \frac{f_X}{n_X(t)(k+\mu)} \left(1 - e^{-(k+\mu)t} \right)$$

where $n_X(t)=X(t)/N(t)$ is the amount of metabolite per cell (mol/cell). When approaching steady state, $(k+\mu)t$>>1, equation (1.22) can be approximated by

$$(1.23) \qquad \varepsilon = 1 - \frac{f_X}{n_X(\infty)(k+\mu)}$$

where $n_X(\infty)$ is the steady state metabolite content per cell. Furthermore, if $n_X(t)$ does not decreases to zero as time increases then from equation (1.11) it follows that ε<<1 for kt>>1 and from equation (1.23) that

$$(1.24) \qquad f_X \approx g_X = n_X(\infty)\left(k+\mu\right)$$

The latter equation reminds us that at steady state the metabolite production (f_X) and consumption (g_X) rates are balanced, and that the consumption rate is equal to $n_X(\infty)$ times the metabolite turn over rate per unit of metabolite k plus the dilution rate μ. The characteristic time τ separating the transient regime from steady state is therefore given by

$$(1.25) \qquad \tau = \frac{1}{k} = \frac{1}{\frac{f_X}{n_X(\infty)} - \mu} \leq \frac{n_X(\infty)}{f_X}$$

This equation can be used to estimate the characteristic transient time of a metabolite given is typical concentration and its production or consumption rate.

Other scenarios

There some cases where the tMFA equation (1.16) applies even though we may not be able to apply the approximation in equation (1.15). For example, if there is only one reaction producing the transient metabolite under consideration, then equation (1.13) is reduced to

(1.26) $$X_i(t) = \delta_{i0} X(0) e^{-kt} + B(t) f_X^*$$

From the latter equation and equation (1.6) one obtains

(1.27) $$x_i = \delta_{i0}\varepsilon + (1-\varepsilon)\varphi_i$$

Equation (1.27) is exactly the tMFA equation (1.16) for the case when there is only one reaction producing the transient metabolite of interest. In this case the tMFA equation (1.16) holds even though the approximation in equation (1.15) may not apply.

2- tMFA model of folate metabolism

Notations

c	D-glucose index
C_i	D-glucose 13C fractions
g	glycine index
G_i	glycine ^{13}C fractions
h	GSH index
H_i	GSH ^{13}C fractions
l	5,10-methylene-THF index
L_i	5,10-methylene-THF ^{13}C fractions
m	methionine index
M_i	L-methionine ^{13}C fractions
n	L-glutamate index
N_i	L-glutamate ^{13}C fractions
r	purines index
R_i	purines ^{13}C fractions
ρ_i	PRPP ^{13}C fractions
s	L-serine index
S_i	L-serine ^{13}C fractions
o	other metabolites index
p	protein index
y	10-formyl-THF index
Y_i	10-formyl-THF ^{13}C fractions
u_i	uptake rate of metabolite i
e_i	export rate of metabolite i
$f_{i,j}$	flux from metabolite i to metabolite j

2.1 Purines balance

The total purine concentration was estimated as the sum of the concentrations of AMP, ADP, ATP, GMP, GDP and GTP. The purines ^{13}C fractions were determined from the ^{13}C fractions of the purines listed above weighted by their concentrations. The purine precursors are 10-formyl-tetrahydrofolate (fTHF), CO_2 and glycine for carbon atoms; aspartate and glutamine for nitrogen atoms. There is no labeling of nitrogen in our experimental setup and therefore, regarding nitrogen, the isotope fractions of aspartate

and glutamine are at steady state. CO_2 can be assumed unlabeled given its high content in the cell culture and therefore its is in steady state. As shown in the main manuscript, we can assume that the glycine isotope fractions are at steady state at the sampling time of 8 hours. The concentration of fTHF is in the $C_{\mathrm{fTHF}}\sim$ 1 μM range and its production/consumption flux in the $f_{\mathrm{fTHF}}\sim$0.1 mM/h (as deduced from the purine synthesis rate (Fig. 2d), resulting in a transient time scale around $C_{\mathrm{fTHF}}/f_{\mathrm{fTHF}}\sim$0.01 h. Therefore we assume that the fTHF isotope fractions are at steady state at the sampling time of 8 hours. The increase in the purines unlabeled fraction 8 hours after MTX (Fig. 2c) indicates that at 8 hours there is still some residual purine from the pool at time zero. Since the purine precursors isotope fractions are at steady state at 8 hours but the purine fractions may still be in a transient dynamics at that time point, we model purines as a transient metabolite. In this case we consider only one metabolic flux, the purine synthesis rate f_{pur}, and therefore $y=f_{pur}/f_{pur}=1$. Furthermore given the stoichiometry of 1 purine per glycine we set $S_{pur}=1$. Following equation (1.16) we write the tMFA equation for purines

(2.1.1) $$R_i = \delta_{i0}\varepsilon + (1-\varepsilon)\varphi_i$$

where φ_i are the ^{13}C fractions of synthesized purines per glycine. Under the assumption that the CO_2 is approximately unlabeled and that PRPP is either fully unlabeled ([U-^{13}C]-L-serine tracing) or fully labeled ([U-^{13}C]-D-glucose tracing), the synthesized purine fractions are given by

(2.1.2)
$$\begin{aligned}
\varphi_0 &= \rho_0 G_0 F_0^2\\
\varphi_1 &= \rho_0 G_0 2F_0F_1\\
\varphi_2 &= \rho_0\left(G_0F_1^2 + G_2F_0^2\right)\\
\varphi_3 &= \rho_0 G_2 2F_0F_1\\
\varphi_4 &= \rho_0 G_2 F_1^2\\
\varphi_5 &= \rho_5 G_0 F_0^2\\
\varphi_6 &= \rho_5 G_0 2F_0F_1\\
\varphi_7 &= \rho_5\left(G_0F_1^2 + G_2F_0^2\right)\\
\varphi_8 &= \rho_5 G_2 2F_0F_1\\
\varphi_9 &= \rho_5 G_2 F_1^2\\
\varphi_{10} &= 0
\end{aligned}$$

Given as input the purines labeling fractions (R_i) and the PRPP labeling fractions (ρ_0, ρ_5), we estimate ε, G_0 and F_0 as the minimum mean squared error solution of the system of equations (2.1.1). Once ε is estimated, we calculate k_{pur} using equation (1.20), $B(t)$ using equation (1.21) with n=2 and f_{pur} using equation (1.12). To this end, we used as input the measured values of $N(0)$, $N(t)$ and $X_{pur}(t)$ at t=8 hours. The concentration of purines at platting, $X_{pur}(0)$, was assumed equal to the average across all replicates and time points in untreated cells.

Based on the rates of purine synthesis and the concentrations of purines reported in the main manuscript, we estimate the purine transient time to be τ=6.86±0.07, 5.92±0.02 and 12.9±0.1 hours in the MCF7, MDA-MB-468 and MDA-MB-231 untreated cells, respectively. Therefore the free purine pool is at a transient state at 8 hours.

2.2 GSH balance

The total GSH concentration was estimated as the sum of the concentrations of reduced and oxidized GSH. The GSH ^{13}C fractions were determined from the ^{13}C fractions of reduced and oxidized GSH weighted by their concentrations. The GSH precursors are cysteine, glutamate and glycine. In the [U-^{13}C]-L-serine experiments cysteine and glutamate are not labeled and therefore their isotope fractions are at steady state (essentially unlabeled). In the [U-^{13}C]-D-glucose experiments cysteine is not labeled and therefore its isotope fractions are at steady state (essentially unlabeled). In the [U-^{13}C]-D-glucose experiments glutamate is labeled and we estimate the time scale to steady state. The concentration of glutamate is close to the C_{glu}~10 mM range and the rate of glutamate consumption at least list its consumption rate for protein synthesis, $g_{\mathrm{glu}}>0.4\,f_P$. The rate of protein synthesis is about 20 mM/h in the studied cells and therefore $g_{\mathrm{glu}}>$ 8 mM/h (Fig. 3f). Based on these number the time scale for the transient dynamics of the glutamate isotope fractions is about $C_{\mathrm{glu}}/g_{\mathrm{glu}}<1$ hour. Therefore we assume that the glutamate isotope fractions are at steady state at the sampling time of 8 hours. As shown in the main manuscript, we can assume that the glycine isotope fractions are at steady state at the sampling time of 8 hours. The increase in the GSH unlabeled fraction 8 hours after MTX (Fig. 4c) indicates that at 8 hours there is still some residual GSH from the pool at time zero. Since the GSH precursors isotope fractions are at steady state at 8 hours but the GSH fractions may still be in a transient dynamics at that time point, we model GSH as a transient metabolite. In this case we consider only one metabolic flux, the GSH synthesis rate f_{GSH}, and therefore $y=f_{\mathrm{GSH}}/f_{\mathrm{GSH}}=1$. Furthermore given the stoichiometry of 1 GSH per glycine we set $S_{\mathrm{GSH}}=1$. Following equation (1.16) we write the tMFA equation for GSH

(2.2.1) $$H_i = \delta_{i0}\varepsilon + (1-\varepsilon)\varphi_i$$

where φ_i are the ^{13}C fractions of synthesized GSH. Under the assumption that cysteine is unlabeled the synthesized unlabeled GSH fraction is given by

(2.2.2) $$\varphi_0 = G_0 N_0$$

Given as input the GSH, glycine and glutamate labeling fractions (H_i, G_i, N_i), we calculate ε from equation (2.2.1). Once ε is estimated, we calculate k_{GSH} using equation (1.20), $B(t)$ using equation (1.21) with n=2 and f_{GSH} using equation (1.12). To this end, we used as input the measured values of $N(0)$, $N(t)$ and $X_{\mathrm{GSH}}(t)$ at t=8 hours. The concentration of GSH at platting, $X_{\mathrm{GSH}}(0)$, was assumed equal the average across all replicates and time points in untreated cells.

Based on the rate of GSH synthesis and the GSH concentration reported in the main manuscript, we estimate the GSH transient time to be τ=10.1±0.4, 13.8±0.2 and 5.1±0.5

hours in the MCF7, MDA-MB-468 and MDA-MB-231 untreated cells, respectively. Therefore the GSH pool is at a transient state at 8 hours.

2.2 Serine ^{13}C fractions balance, [U-^{13}C]-L-serine tracing

Intracellular serine has a concentration in the mM range and the net flux of serine production/consumption is in the mM/h range, resulting in a typical transient scale of about 1 hour. Therefore serine can be treated as a steady state metabolite at the 8 hours sampling point. Serine can be produced from glucose (f_{cs}), imported from the media (u_s) or produced from the reverse flux of SHMT (f_{gs}). The serine ^{13}C fractions satisfy the flux balance equations

(2.2.1) $$S_0\left(u_s + f_{cs} + f_{gs}\right) = f_{cs} + L_0 G_0 f_{gs}$$

(2.2.2) $$S_1\left(u_s + f_{cs} + f_{gs}\right) = L_1 G_0 f_{gs}$$

(2.2.3) $$S_2\left(u_s + f_{cs} + f_{gs}\right) = L_0 G_2 f_{gs}$$

(2.2.4) $$S_3\left(u_s + f_{cs} + f_{gs}\right) = u_s + L_1 G_2 f_{gs}$$

These are the MFA equations for serine. Since the ^{13}C fractions sum to 1, this is a system of three equations with three variables, f_{cs}, f_{gs} and L_0. A convenient way to solve this system of equations is to introduce the auxiliary variables

(2.2.5) $$x = \frac{f_{cs}}{f_{cs} + u_s + f_{gs}}$$

(2.2.6) $$y = \frac{f_{gs}}{f_{cs} + u_s + f_{gs}}$$

(2.2.7) $$z = \frac{L_0 f_{gs}}{f_{cs} + u_s + f_{gs}}$$

With this change of variables equations (2.2.1)-(2.2.3) can be rewritten as

(2.2.8) $$x + G_0 y = S_0$$

(2.2.9) $$G_0 y - G_0 z = S_1$$

(2.2.10) $$G_2 z = S_2$$

The solution of the linear system of equations (2.2.8)-(2.2.10) is

(2.2.11) $$x = S_0 - \frac{G_0}{G_2} S_2$$

(2.2.12) $$y = \frac{S_1}{G_0} + \frac{S_2}{G_2}$$

(2.2.13) $$z = \frac{S_2}{G_2}$$

Expressing f_{cs}, f_{gs} and $L_0 f_{gs}$ as the product of x, y and z, respectively, times $f_{gs}+u_s+f_{gs}$ and substituting the result into equation (2.2.4) we obtain

(2.2.14) $$f_{cs} + u_s + f_{gs} = \frac{u_s}{S_3 - G_2(y - z)}$$

Finally, from equations (2.2.5)-(2.2.7) we obtain

(2.2.15) $$f_{cs} = \frac{S_0 - \frac{G_0}{G_2}S_2}{S_3 - \frac{G_2}{G_0}S_1}u_s$$

(2.2.16) $$f_{gs} = \frac{\frac{S_1}{G_0} + \frac{S_2}{G_2}}{S_3 - \frac{G_2}{G_0}S_1}u_s$$

(2.2.17) $$L_0 = \frac{\frac{G_0}{G_2}}{\frac{S_1}{S_2} + \frac{G_0}{G_2}}$$

The serine production/consumption flux balance satisfy the equation

(2.2.18) $$u_s + f_{cs} + f_{gs} = f_{sg} + f_{sp} + f_{so}$$

where f_{sg} is the forward rate of SHMT converting serine to glycine, f_{sp} is the rate of serine consumption for protein synthesis and f_{so} is the rate of serine consumption to other metabolites. From this equation we obtain

(2.2.19) $$f_{so} = u_s + f_{cs} + f_{gs} - f_{sg} - f_{sp}$$

In addition we define the same rate relative to the serine consumption rate by protein synthesis

(2.2.20) $$\Delta_{ser} = \frac{f_{so}}{f_{sp}}$$

2.3 Serine 13C fractions balance, [U-^{13}C]-D-glucose tracing

In this context the serine ^{13}C fractions satisfy the flux balance equations

(2.3.1) $$S_0\left(u_s + f_{cs} + f_{gs}\right) = u_s + L_0 G_0 f_{gs}$$

(2.3.2) $$S_1\left(u_s + f_{cs} + f_{gs}\right) = L_1 G_0 f_{gs}$$

(2.3.3) $$S_2\left(u_s + f_{cs} + f_{gs}\right) = L_0 G_2 f_{gs}$$

(2.3.4) $$S_3\left(u_s + f_{cs} + f_{gs}\right) = f_{cs} + L_1 G_2 f_{gs}$$

Since the ^{13}C fractions sum to 1, this is a system of three equations with three variables, f_{cs}, f_{gs} and L_0. A convenient way to solve this system of equations is to introduce the auxiliary variables (2.2.5)-(2.2.7). With this change of variables equations (2.3.1)-(2.3.4) can be rewritten as

(2.3.5) $$G_0 y - G_0 z = S_1$$

(2.3.6) $$G_2 z = S_2$$

(2.3.7) $$x + G_2 y - G_2 z = S_3$$

The solution of the linear system of equations (2.3.5)-(2.3.7) is

(2.3.8) $$x = S_3 - \frac{G_2}{G_0} S_1$$

(2.3.9) $$y = \frac{S_1}{G_0} + \frac{S_2}{G_2}$$

(2.3.10) $$z = \frac{S_2}{G_2}$$

Expressing f_{cs}, f_{gs} and $L_0 f_{gs}$ as the product of x, y and z, respectively, times u_s+f_{gs}+ f_{gs} and substituting the result into equation (2.3.1) we obtain

(2.3.11) $$u_s + f_{cs} + f_{gs} = \frac{u_s}{S_3 - G_0 z}$$

Finally, from equations (2.3.8)-(2.3.10) we obtain

(2.3.12) $$f_{cs} = \frac{S_3 - \frac{G_2}{G_0} S_1}{S_0 - \frac{G_0}{G_2} S_2} u_s$$

(2.3.13) $$f_{gs} = \frac{\frac{S_1}{G_0} + \frac{S_2}{G_2}}{S_0 - \frac{G_0}{G_2} S_2} u_s$$

(2.3.14) $$L_0 = \frac{\frac{G_0}{G_2}}{\frac{S_1}{S_2} + \frac{G_0}{G_2}}$$

Based on the net rate of serine production (media uptake, from glucose, from reverse SHMT) and the serine concentration reported in the main manuscript, we estimate the serine transient time to be τ=0.13±0.01, 0.18±0.02 and 0.15±0.02 hours in the MCF7, MDA-MB-468 and MDA-MB-231 untreated cells, respectively. Therefore serine is at steady state at 8 hours.

2.4.Glycine balance

Intracellular glycine has a concentration in the mM range and the net flux of glycine production/consumption is in the mM/h range, resulting on a typical transient scale of about 1 hour. Therefore glycine can be treated as a steady state metabolite at the 8 hours sampling point. Glycine can be imported from the media (u_g), produced from serine (f_{sg}), produced from 5,10-methylene-THF and CO_2 via reverse GCS (f_{lg}), or produced from other metabolites such as choline (f_{og}). The glycine ^{13}C fractions satisfy the flux balance equations

(2.4.1) $$G_0 g_G = u_g + \left(S_0 + S_1 - S_{010}\right) f_{sg} + L_0 f_{\mathrm{lg}} + f_{og}$$

(2.4.2) $$G_1 g_G = \left(S_{010} + S_{011}\right) f_{sg} + L_1 f_{\mathrm{lg}}$$

(2.4.3) $$G_2 g_G = \left(S_2 + S_3 - S_{011}\right) f_{sg}$$

where g_G is the net rate of glycine consumption, S_{010} is the fraction of M+1 serine where the ^{13}C atom is in the second position, and S_{011} is the fraction of M+2 serine where the ^{13}C atoms are in the second and third position. We note that G_1 is in general very small ($G_1 << G_0+G_2$) in our samples. In our experimental setup ([U-^{13}C]-D-glucose or [U-^{13}C]-L-serine) S_{010} and S_{011} can only be obtained from G_1 and therefore $S_{010} << S_0+S_1$ and $S_{011} << S_2+S_3$. Thus from (2.4.3) it follows that

(2.4.5) $$f_{sg} = \frac{G_2}{\left(S_2 + S_3 - S_{011}\right)} g_G \approx \frac{G_2}{\left(S_2 + S_3\right)} g_G$$

Substituting (2.4.5) into (2.4.2) we obtain

(2.4.6) $$f_{\mathrm{lg}} = \frac{1}{L_1}\left[G_1 - \frac{G_2}{S_2 + S_3}\left(S_{010} + S_{011}\right)\right] g_G$$

$S_{010}+S_{011}$ can be calculated from the balance of serine

(2.4.7) $$\left(S_{010} + S_{011}\right) g_S = G_1 f_{gs}$$

resulting in

(2.4.8) $$\left(S_{010} + S_{011}\right) = G_1 \frac{f_{gs}}{g_S}$$

Substituting (2.4.8) in (2.4.6) we obtain

(2.4.9) $$f_{lg} = \frac{G_1}{L_1}\left[G_1 - \frac{G_2}{S_2+S_3}\frac{f_{gs}}{g_S}\right]g_G$$

Finally, using flux balance $g_G=f_G=u_g+f_{sg}+f_{lg}+f_{og}$ we obtain

(2.4.10) $$f_{og} = g_G - u_g - f_{sg} - f_{lg}$$

In addition we define the same rate relative to the glycine consumption rate by protein synthesis

(2.4.11) $$\Delta_{gly} = \frac{f_{og}}{f_{gp}}$$

We calculate f_{sg}, f_{lg} and f_{og} using equations (2.4.5), (2.4.9) and (2.4.10), assuming that the glycine consumption rate is given by $g_G=e_g+f_{gs}+f_{gr}+f_{gh}$, where e_g is the glycine export rate (as measured), f_{gs} is the reverse SHMT rate (calculated in the previous section), $f_{gr}=f_{pur}$ is the glycine consumption rate for purine synthesis, and $f_{gh}=f_{\mathrm{GSH}}$ is the glycine consumption rate for GSH synthesis. We also assume that $g_S=f_S=u_s+f_{cs}+f_{gs}$.

Based on the calculated glycine production/consumption rate ($f_G=g_G$) and the glycine concentration reported in the main manuscript, we estimate the glycine transient time to be τ=0.34±0.02, 0.36±0.06 and 0.7±0.2 hours in the MCF7, MDA-MB-468 and MDA-MB-231 untreated cells, respectively. Therefore glycine is at a steady state at 8 hours.

2.5 Methionine balance

Methionine can be imported from the media (u_m) and produced by methionine synthase (f_{lm}). Methionine synthase transfer a methyl group from 5-methyl-THF to homocysteine. In turn, 5-methy-THF is produced from 5,10-methylene-THF by 5,10-methylene-THF reductase. The methionine M_0 fraction satisfy the flux balance equation

(2.5.1) $$M_0\left(u_m + f_{lm}\right) = u_m + L_0 f_{lm}$$

From this equation we obtain

(2.5.2) $$f_{lm} = \frac{1-M_0}{M_0 - L_0}u_m$$

The methionine production/consumption flux balance satisfy the equation

(2.5.3) $$u_m + f_{lm} = f_{mp} + f_{mo}$$

From this equation we obtain

(2.5.4) $$f_{mo} = u_m + f_{lm} - f_{mp}$$

In addition we define the same rate relative to the serine consumption rate by protein synthesis

(2.5.5) $$\Delta_{met} = \frac{f_{mo}}{f_{mp}}$$

3 Thymidylate synthesis rate

The thymidylate synthesis rate of a proliferating cell is given by $f_{dTMP}=\mu b x_T/V$, where $\mu=\ln 2/T_D$ is the proliferating rate, T_D is the doubling time, b is the number of DNA bases in the cell genome and x_T is the fraction of base T in the genome. In general x_T~1/4. For cancer cells T_D~48 hours, b~15 fmol/cell, and V~3 pL [1]. Based on these numbers we obtain f_{dTMP}~0.02 mM/h.

4 Exchange fluxes

In this section we focus our attention on a given extratracellular metabolite X with a time dependent total content $X(t)$ (mol/cell culture). The transient dynamics of X follow the equation

(4.1) $$\dot{X}(t) = N(t)e_X - N(t)u_X$$

where $N(t)$ is the number of cells, e_X is the export rate per cell and u_X is the uptake rate per cell. Integrating equation (4.1) we obtain

(4.2) $$u_X - e_X = \frac{X(0)-X(t)}{A(t)}$$

where

(4.3) $$A(t) = \int_0^t dt' N(t')$$

$A(t)$ can be estimated by approximating the integral by the trapezoidal rule with n intervals of uniform length $\Delta t=t/n$, obtaining

(4.4) $$A(t) = \frac{\Delta t}{2}\sum_{i=1}^{n}\left[N(t_{i+1}) + N(t_i)\right]$$

Measuring $N(t)$ at the i=1,…,n time points we can estimate $A(t)$ using equation (4.4). We calculate $A(t)$ using equation (4.4) with n=2, using as input the measured values of $N(0)$ and $N(t)$ at t=8 hours.

5 Correcting for ^{13}C natural abundance

^{13}C can be found in nature at a 1.1%. Because of that the measured ^{13}C fractions are slightly higher than what expected if all unlabelled nutrients where fully composed of ^{12}C. Given a metabolite with n carbon atoms, if X_i are the average ^{13}C fractions assuming that the natural occurrence of ^{13}C is zero, q is the probability that a carbon atom is ^{13}C in nature (q=0.011), and Y_i are the average ^{13}C fractions taking into account the natural occurrence of ^{13}C (the expected value of the measured ^{13}C fractions), then

(5.1) $$Y_i = \sum_{j=0}^{i} X_i \begin{pmatrix} n-i \\ i-j \end{pmatrix} q^{i-j}(1-q)^{n-i}$$

From this equation we obtain

$$(5.2) \qquad X_i = \begin{cases} \dfrac{1}{(1-q)^n} Y_0 & i = 0 \\ \dfrac{1}{(1-q)^{n-i}} \left[Y_i - \sum_{j=0}^{i-1} X_j \begin{pmatrix} n-i \\ i-j \end{pmatrix} q^{i-j} (1-q)^{n-i} \right] & 1 \le i \le n \end{cases}$$

Using (5.2) we can recursively calculate the ^{13}C fractions X_i after removing the natural occurrence of ^{13}C.

References

1. Dolfi SC, Chan LL, Qiu J, Tedeschi PM, Bertino JR, Hirshfield KM, Oltvai ZN, Vazquez A: **The metabolic demands of cancer cells are coupled to their size and protein synthesis rates**. *Cancer & metabolism* 2013, **1**(1):20.

Supplementary figures

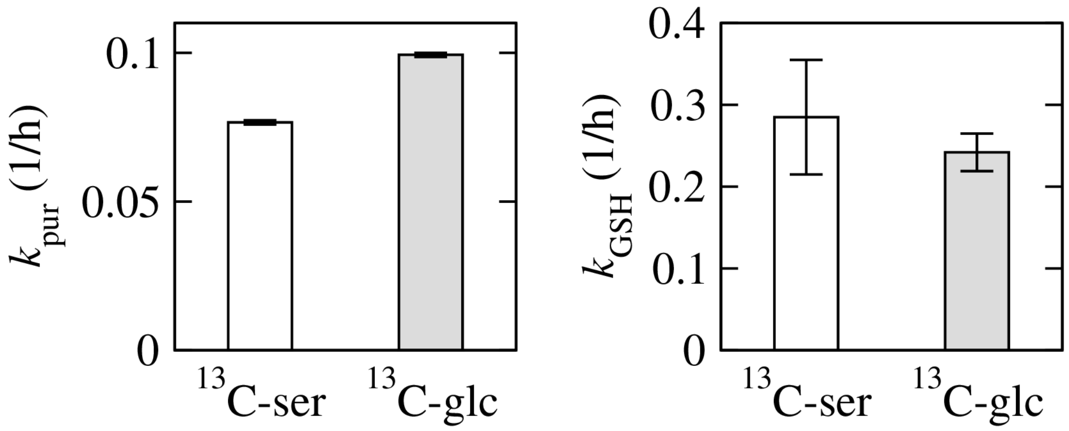

Supplementary Figure 1: Estimated turnover rates per unit of metabolite for purines and glutathione in MDA-MB-231 cells, using either [U-^{13}C]-L-serine (white bars) or [U-^{13}C]-D-glucose (grey bars).

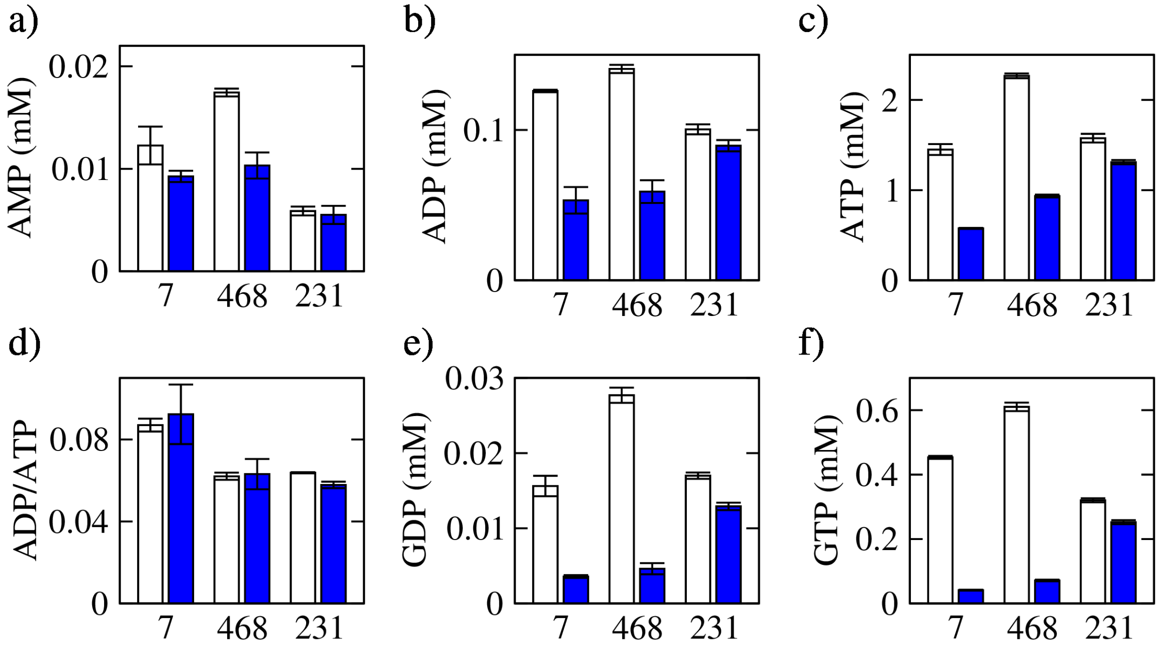

Supplementary Figure 2: Purine nucleotides concentration in untreated cells (open white bars) and cells treated with 100 nM MTX (solid blue bars). GMP levels were below detection limits.

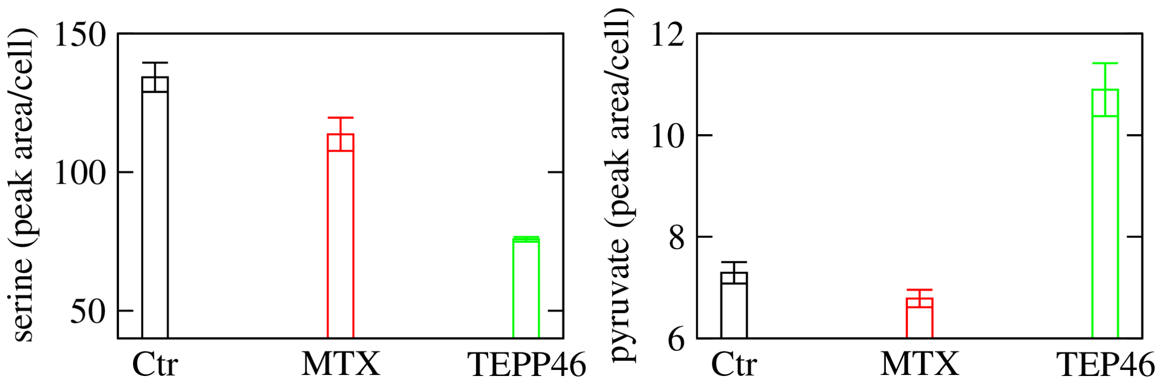

Supplementary Figure 5: Changes in the levels of serine and pyruvate after treatment with MTX or TEPP-46, relative to their values in untreated cells (Ctr).

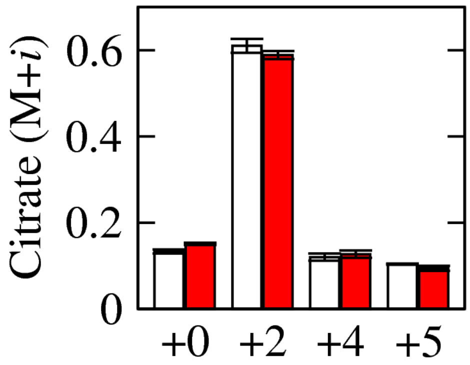

Supplementary Figure 7: Major citrate isotope fractions in MDA-MB-231 cells growing in a medium containing [U-^{13}C]-D-glucose, untreated (open white bars) or treated with 100 nM atorvastatin (filled red bars).
